# Disentangling the Roles of RIM and Munc13 in Synaptic Vesicle Localization and Neurotransmission

**DOI:** 10.1523/JNEUROSCI.1922-20.2020

**Published:** 2020-12-02

**Authors:** Fereshteh Zarebidaki, Marcial Camacho, Marisa M. Brockmann, Thorsten Trimbuch, Melissa A. Herman, Christian Rosenmund

**Affiliations:** Institute of Neurophysiology and NeuroCure Cluster of Excellence, Charité-Universitätsmedizin, Berlin, 10117, Germany

**Keywords:** active zone, electron microscopy, Munc13, RIM, synaptic transmission, synaptic vesicle

## Abstract

Efficient neurotransmitter release at the presynaptic terminal requires docking of synaptic vesicles to the active zone membrane and formation of fusion-competent synaptic vesicles near voltage-gated Ca^2+^ channels. Rab3-interacting molecule (RIM) is a critical active zone organizer, as it recruits Ca^2+^ channels and activates synaptic vesicle docking and priming via Munc13-1. However, our knowledge about Munc13-independent contributions of RIM to active zone functions is limited. To identify the functions that are solely mediated by RIM, we used genetic manipulations to control RIM and Munc13-1 activity in cultured hippocampal neurons from mice of either sex and compared synaptic ultrastructure and neurotransmission. We found that RIM modulates synaptic vesicle localization in the proximity of the active zone membrane independent of Munc13-1. In another step, both RIM and Munc13 mediate synaptic vesicle docking and priming. In addition, while the activity of both RIM and Munc13-1 is required for Ca^2+^-evoked release, RIM uniquely controls neurotransmitter release efficiency. However, activity-dependent augmentation of synaptic vesicle pool size relies exclusively on the action of Munc13s. Based on our results, we extend previous findings and propose a refined model in which RIM and Munc13-1 act in overlapping and independent stages of synaptic vesicle localization and release.

**SIGNIFICANCE STATEMENT** The presynaptic active zone is composed of scaffolding proteins that functionally interact to localize synaptic vesicles to release sites, ensuring neurotransmission. Our current knowledge of the presynaptic active zone function relies on structure-function analysis, which has provided detailed information on the network of interactions and the impact of active zone proteins. Yet, the hierarchical, redundant, or independent cooperation of each active zone protein to synapse functions is not fully understood. Rab3-interacting molecule and Munc13 are the two key functionally interacting active zone proteins. Here, we dissected the distinct actions of Rab3-interacting molecule and Munc13-1 from both ultrastructural and physiological aspects. Our findings provide a more detailed view of how these two presynaptic proteins orchestrate their functions to achieve synaptic transmission.

## Introduction

At presynaptic terminals, neurotransmitter release occurs at specific regions known as active zones (AZ). AZs are morphologically characterized by a complex network of proteins (Gundelfinger et al., 2003). The proteins that constitute the cytomatrix of the AZ (CAZ) play a central role in neurotransmitter release by localizing and clustering Ca^2+^ channels, and by regulating the stages of the synaptic vesicle (SV) cycle, such as tethering, docking, priming, and fusion (Sudhof, 2012). Among these functions, dissecting the role of specific molecular components in different steps of the SV cycle has presented technical challenges: (1) vesicle tethering and docking can only be distinguished from the downstream physiological events of vesicle priming and release by ultrastructure (Verhage and Sorensen, 2008); and (2) the individual protein components of the CAZ may contribute to more than one stage of neurotransmitter release (Ackermann et al., 2015).

Because CAZ proteins are densely positioned at the AZ, with many putative interaction sites between them, disentangling the functions of individual AZ proteins requires a multitude of genetic ablation and rescue analyses. The AZ protein core consists of Rab3-interacting molecule (RIM), Munc13, RIM-binding protein (RIM-BP), ELKS, α-Liprins, Piccolo, and Bassoon (Schoch and Gundelfinger, 2006; Sudhof, 2012). RIM is of particular interest, as investigations have shown its extensive interaction and regulatory effect on other AZ proteins. The two mammalian genes, *Rims1* and *Rims2*, synthesize five RIM isoforms, RIM1α/β, and RIM2α/β/γ, whose domain content varies their interactions with Rab3, Munc13, and Ca^2+^ channels (Kaeser et al., 2008, 2012). Genetic deletion of both *Rim* genes shows that α- and β-RIMs directly interact with Ca^2+^ channels, and indirectly, via RIM-BP, target Ca^2+^ channels to release sites, as the absence of RIMs leads to an impaired presynaptic Ca^2+^ channel function, Ca^2+^ channel-vesicle coupling, Ca^2+^-evoked release, and vesicular release probability (Hibino et al., 2002; Kiyonaka et al., 2007; Han et al., 2011, 2015; Kaeser et al., 2011; Graf et al., 2012; Hirano et al., 2017). In addition, α-RIMs and RIM1β promote the activity of the priming factor, Munc13, by interfering with priming-impeding Munc13 homodimerization (Betz et al., 2001; Deng et al., 2011; Camacho et al., 2017) and by recruiting Munc13 to the presynaptic AZ (Andrews-Zwilling et al., 2006). As Munc13s play a key role in determining the number of release sites and fusion-competent vesicles (Augustin et al., 1999a,b; Sakamoto et al., 2018; Bohme et al., 2019), it is challenging to deconvolve the direct role of RIM1/2 in the SV cycle from its role in stabilizing Munc13 at the AZ.

RIM also ensures the integrity of AZ scaffolds with its large molecular interactors, such as RIM-BP (Acuna et al., 2016; Wu et al., 2019) and ELKS (S. S. Wang et al., 2016). RIM's role in determining AZ scaffold integrity manifest on an ultrastructural level. Cryo-electron tomographic experiments revealed fewer tethered vesicles and a lower vesicle density in RIM1α KO synaptosomes, suggesting a distinct role for RIM in vesicle localization close to the AZ membrane (Fernández-Busnadiego et al., 2013). This effect may be mediated by the dynamic tripartite complex of α-RIMs, Rab3, and Munc13 that targets vesicles to the release sites (Dulubova et al., 2005). Therefore, comparative ultrastructural evidence in RIM and Munc13-deficient neurons is necessary to dissect the Munc13-dependent and -independent roles of RIM in SV localization and docking at the AZ.

In our study, we aimed to disentangle RIM's direct role in the SV cycle at the AZ from its role in enabling the function of Munc13. Using genetic deletions, we systematically compared murine hippocampal synapses lacking RIM1/2 and/or Munc13-1, the predominant isoform of Munc13 in mouse CNS. Comparative analysis of neurotransmission by electrophysiology, as well as ultrastructural analysis by electron microscopy, revealed a Munc13-1-independent contribution of RIM to vesicular release probability and SV localization. Together, our results provide a deeper understanding of key AZ proteins function individually and in concert to ensure efficient neurotransmission.

## Materials and Methods

### 

#### 

##### Animals and maintenance

All animal experiments and maintenance were approved by Animal Welfare Committee of Charité-Universitätsmedizin Berlin and the Berlin state government agency for Health and Social Services (license no. T 0220/09). The *RIM1^flox^*/*RIM2^flox^* and *RBP1^flox^*/*RBP2^flox^* mouse line, a gift from the Thomas C. Südhof laboratory (Acuna et al., 2016), was crossed with C57BL/6N mice to generate the *RIM1^flox^*/*RIM2^flox^* mouse line (called RIM1/2*^flox^*). The RIM1/2*^flox^* animals were interbred, and the offspring at postnatal (P) days 0-2 were used to obtain the RIM1/2 control and conditional double KO (cDKO) neurons. *Munc13-1*^−/−^ and *Munc13-1*^+/+^ littermates at embryonic (E) day 18 were obtained by interbreeding of *Munc13-1*^+/−^ mouse line on an FVB/N background. Here, we referred to *Munc13-1*^+/+^ as Munc13-1 WT and to *Munc13-1*^−/−^ as Munc13-1 KO. Moreover, the mouse line of *Munc13-2*^−/−^/*Munc13-1*^+/−^ on an FVB/N background (Camacho et al., 2017) was interbred to produce *Munc13-2*^−/−^/*Munc13-1*^+/+^ animals at E18. Gender of the animals used for experimentation was not distinguished.

##### Neuronal cultures and lentiviral infections

Micro-islands and continental astrocyte feeder layers were generated from cortices of P0-P1 C57BL/6N mice 2 weeks before the neuronal culture preparations as described previously (Arancillo et al., 2013). To generate neuronal cultures, the hippocampi from either P0-P2 RIM1/2*^flox^* pups, or Munc13-1 WT, Munc13-1 KO, Munc13-2 KO embryos at E18 were dissected. After enzymatic digestion with papain solution (Worthington), neurons were mechanically dissociated. Neurons were counted and seeded on astrocytic feeder layers in Neurobasal-A medium (Invitrogen) supplemented with B-27 (Invitrogen), 50 IU/ml penicillin, and 50 μg/ml streptomycin (Invitrogen). For autaptic cultures, 3500 cells were seeded on coverslip glasses (30 mm) containing an astrocytic micro-island pattern to perform immunocytochemistry and electrophysiological recordings. For the mass cultures, 100,000 cells were plated on 6-well plates with astrocytic feeder layers to perform Western blot, and 100,000 cells were seeded on sapphire glasses (6 mm) with an astrocytic feeder layer for high-pressure freezing experiments.

After 1-2 DIV, neurons were transduced with lentiviral particles. RIM1/2 control and cDKO were generated from RIM1/2*^flox^* hippocampal neurons infected with lentivirus containing inactive and active Cre recombinase tagged with EGFP (Kaeser et al., 2011). Short hairpin RNA (shRNA) target sequence (5′-GCCTGAGATCTTCGAGCTTAT-3′) for Munc13-1 was previously described (Deng et al., 2011). The shRNA sequence was cloned into a lentiviral shuttle vector that controlled its expression via a U6 promoter. A further human synapsin-1 promoter controlled the expression of an NLS-RFP protein to label infected neurons. The viral production was performed by the Viral Core Facility of the Charité-Universitätsmedizin Berlin. The titer of Munc13-1 KD shRNA was assessed by qPCR using LV900 Lentivirus Titration Kit (Applied Biological Materials).

##### Electrophysiology in the hippocampal autaptic culture

Whole-cell voltage-clamp recordings were performed on autaptic hippocampal neurons at DIV 15-20. Single neurons on micro-islands were selected and recorded at room temperature using Multiclamp 700B amplifier (Molecular Devices) under the control of Clampex 10.5 software. Axon Digidata 1550 digitizer (Axon Instruments) was used for data acquisition at 10 kHz sample rate with a low-pass Bessel filter at 3 kHz. Borosilicate glass pipettes with resistance between 2 and 4 mΩ were pulled with a micropipette puller device (Sutter Instruments). The pipettes were filled with intracellular solution containing the following (in mm): 136 KCl, 17.8 HEPES, 1 EGTA, 4.6 MgCl_2_, 4 Na_2_ATP, 0.3 Na_2_GTP, 12 creatine phosphate, and 50 U ml^−1^ phosphocreatine kinase (∼300 mOsm, pH 7.4). The extracellular solution contained the following (in mm): 140 NaCl, 2.4 KCl, 10 HEPES, 10 glucose, 2 CaCl_2_, and 4 MgCl_2_ (∼300 mOsm, pH 7.4) and was permanently exchanged with a fast flow system. During the recordings, the access resistance was compensated at 70%. The neurons with <10 mΩ series resistance were used for the recordings. The EPSCs were induced by 2 ms depolarization from holding potential of −70 to 0 mV. Extracellular solution containing 3 μm NBQX was used to distinguish glutamatergic from GABAergic neurons. To identify spontaneous events, traces recorded at a holding potential of −70 mV were filtered at 1 kHz, and mEPSCs were detected by a template algorithm in Axograph X (Axograph Scientific). False-positive events were excluded by subtracting events detected from traces in the presence of NBQX. The readily releasable pool (RRP) of SVs was estimated by application of 500 mm sucrose solution for 5 s. The charge of the transient response component was used to determine the RRP size (Rosenmund and Stevens, 1996). The vesicular release probability (*P*_vr_) was determined by dividing the EPSC charge by the sucrose-induced charge. Paired-pulse protocol was induced by two sequential action potentials (APs) with 25 ms interstimulus interval. Paired-pulse ratio was calculated by dividing the amplitude of the second EPSC by the amplitude of the first EPSC. The RRP augmentation was assessed by calculating the ratio of the sucrose-induced charge 2 s after 10 Hz stimulation with 50 APs to the baseline sucrose-induced charge. Electrophysiological recordings were analyzed using Axograph X (Axograph Scientific). Standard Hill equation *Y* = *M*/[1 + (*K*_d_/*X*)*^n^*] was performed to fit the dose–response relationship of electrophysiological parameters as a function of the protein expression (Arancillo et al., 2013). In the equation, *Y* is response amplitude, *X* is Munc13-1 relative expression to VGLUT1, *M* is the maximum response, *K*_d_ is the dissociation constant, and *n* is cooperativity.

##### High-pressure freezing and transmission electron microscopy

The 6 mm carbon-coated sapphire glass-containing neurons (DIV 15-16) were frozen in high-pressure freezing device (Leica Microsystems, EM ICE or HPM100). After freezing, the samples were transferred into a liquid nitrogen chamber. Each sample was moved inside the AFS2 automated freeze-substitution device (Leica Microsystems) containing cryovials for each sapphire with the following solution: 1% osmium tetroxide, 1% glutaraldehyde, 1% ddH_2_O in anhydrous acetone. The AFS2 device was programmed for a 2 d protocol with a stepwise heating starting at −90°C for 4-5 h, −90°C to −20°C for 14 h, −20°C for 12 h, and −20°C to 20°C for 8 h. Subsequently, samples were washed with anhydrous acetone and were treated for 1 h with 0.1% uranyl acetate to get contrast enhancement. In the last step, samples were embedded in EPON and baked at 60°C for 48 h to polymerize. The serial sectioning was performed with an ultramicrotome (Leica Microsystems) to obtain 40 nm sample thickness. Samples were collected in formvar-coated single-slot grids (Science Services). Just before the imaging, samples were contrasted for 3-5 min in 2% uranyl acetate and for 30 s in 0.3% lead citrate in ddH_2_O. The images were obtained with a FEI Tecnai G20 transmission electron microscopy operating at 200 keV. Synapses were selected based on detectable postsynaptic density (PSD). For each experiment, ∼50 profiles per group were imaged with (2048 × 2048 pixels) CCD camera (Olympus) at 0.73 nm pixel size. The analysis was performed blind using ImageJ software and MATLAB. AZ length was defined as the membrane opposite to the PSD. SVs were identified as visible circular membranes structures with a diameter of ∼25-50 nm in a single plane of a 2D micrograph. The visualization of membranes was aided by increasing contrast. To avoid bias, the analysis for each experiment was performed by one person who was blind to the experimental groups. The SVs that were directly in contact with the AZ membrane were marked as docked SVs. SVs not directly in contact with the AZ membrane, but residing within 100 nm of the AZ, were identified and their shortest distance to the AZ membrane was measured (Watanabe et al., 2013). To facilitate the presentation and discussion of our findings with regard to changes in SV localization as a function of RIM/Munc13-1 protein level manipulations, we used the term “proximal” to refer to the SVs up to 20 nm from the AZ membrane, whereas the SVs at >20-100 nm from the AZ were referred to as “distal.”

##### Immunostaining and quantification

Autaptic neurons at DIV 15-20 were fixed for 10 min in 4% PFA, permeabilized with 0.1% PBS-Tween-20 solution and blocked with 5% normal donkey serum. Rabbit anti-VGLUT1 (SYSY 135302) and Guinea-pig anti-Munc13-1 (SYSY 126104) primary antibodies were used for coimmunostaining to detect Munc13-1 presynaptic expression. The primary antibodies were labeled with AlexaFluor-488 anti-rabbit IgG, as well as AlexaFluor-647 anti-guinea pig both in donkey serum (Jackson ImmunoResearch Laboratories), respectively. Single neurons on the astrocytic micro-islands were imaged using a Nikon scanning confocal microscopy A1Rsi with a × 60 oil immersion objective. The *z*-series images at 0.3 µm depth were obtained at equal exposure times with 1024 × 1024 pixels resolution and at the pixel size of 0.2 µm. The analysis was performed blind in ImageJ software by drawing ROIs of 50 synapses per neuron. ROIs were defined by staining for the SV marker VGLUT1. Munc13-1 fluorescence intensity for each synapse was divided to the corresponding fluorescence intensity of VGLUT1; ∼30 neurons per group were analyzed in three independent cultures, and the data were normalized to the control of each experiment.

##### Western blot

Protein lysates for Western blot were obtained from mass culture neurons at DIV 15-20. The lysis solution contained 50 mM Tris/HCl, pH 7.6, 150 mM NaCl, 1% Nonidet P-40, 0.5% sodium deoxycholate, and 4% of Complete Protease Inhibitor (Roche Diagnostics). Lysed cells were scrubbed from plates and centrifuged to remove the debris from the protein-content supernatant; 30 µg of proteins was loaded to SDS-polyacrylamide gel, separated after electrophoresis, and transferred to nitrocellulose paper (Bio-Rad). The proteins were labeled with primary antibodies during overnight incubation at 4°C. The mouse anti-Tubulin III antibody (Sigma Millipore, T8660) was used to label Tubulin III as a loading control. Rabbit anti-Munc13-1 (SYSY 126103) or rabbit anti-RIM1/2 (SYSY 140203) was used to label Munc13-1 and RIM1/2, respectively. Goat IgG HRP-conjugated antibody (Jackson ImmunoResearch Laboratories) was used for the labeling of primary antibodies and was detected by ECL solution (GE Healthcare Biosciences). To quantify the proteins' expression level, the ratio of either Munc13-1 or RIM1/2 signal density to the corresponding Tubulin III signal density was normalized to the control group in three independent cultures.

##### Statistical analysis

**Figure 1.**
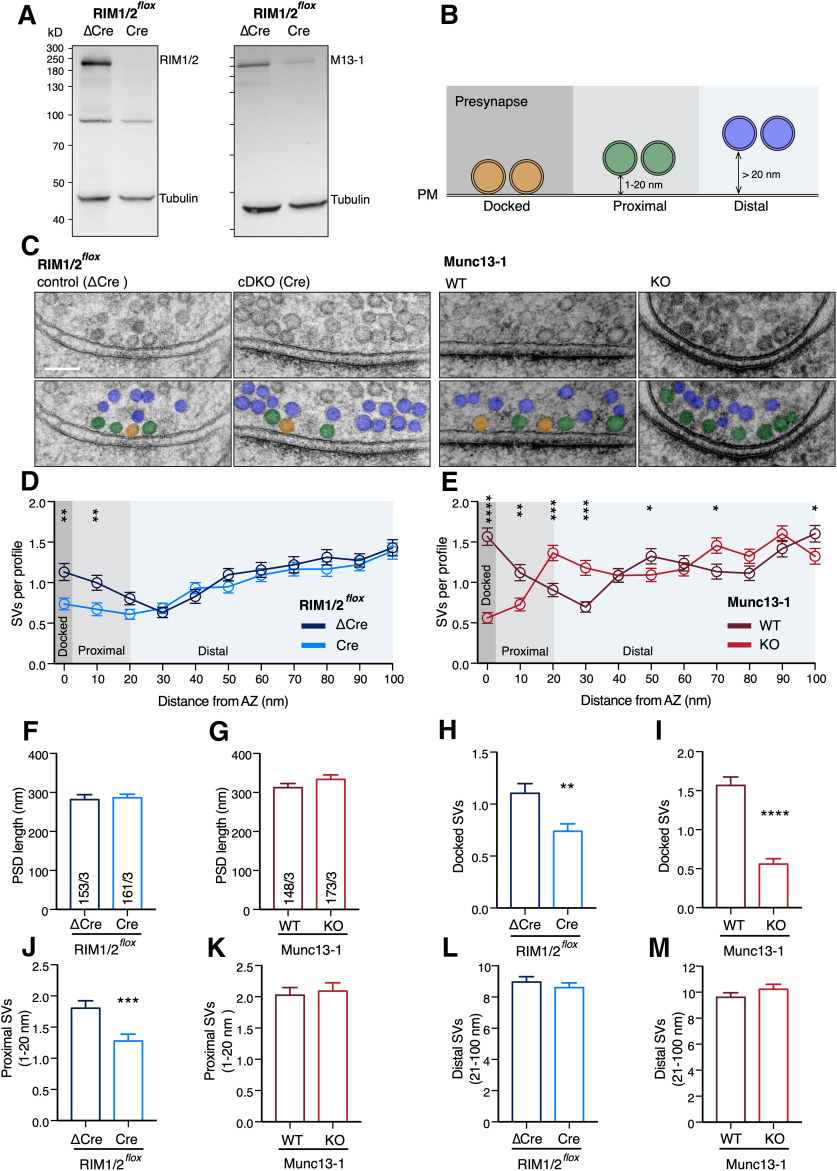
RIM1/2 and Munc13-1 differentially affect SV distribution and docking. ***A***, Immunoblots of lysates from RIM1/2*^flox^* hippocampal neurons infected with ΔCre (control) and Cre recombinase (cDKO) detecting RIM1/2 (left) and Munc13-1 (right) expression. Tubulin expression was used as a loading control. Left, Markers designate the molecular weight. ***B***, Diagram represents the analysis of SVs based on their distance from AZ membrane. P.M., plasma membrane. ***C***, Example TEM images displaying the presynaptic area of RIM1/2 control and cDKO (left), as well as Munc13-1 WT and KO (right). Top, Raw images. Bottom, Vesicles are color-coded according to their distance to the AZ membrane: docked SVs (orange), proximal SVs (green), and distal SVs (blue). Scale bar, 100 nm. Additional example pictures are represented in Extended Data Figure 1-2. ***D***, ***E***, Plots represent the number of SVs as a function of distance from the AZ membrane for RIM1/2 cDKO (***D***) and Munc13-1 KO (***E***) synapses, compared with their corresponding control (binned to 10 nm). ***F***-***M***, Bar plots represent the mean PSD length (***F***,***G***), docked SVs (***H***,***I***), proximal SVs (1-20 nm) (***J***,***K***), and distal SVs (21-100 nm) (***L***,***M***) for RIM1/2 cDKO and Munc13-1 KO synapses compared with their corresponding controls. The data are obtained from the same experimental settings for all the electron microscopy analysis (RIM1/2 control: 153/3 and RIM1/2 cDKO: 161/3; Munc13-1 WT: 148/3 and Munc13-1 KO: 173/3) indicated in ***F*** and ***G***. The numbers are obtained from three independent cultures. Values indicate mean ± SEM. *p ≤ 0.05; **p ≤ 0.01; ***p ≤ 0.001; ****p ≤ 0.0001; nonparametric *t* test, followed by Mann–Whitney test. For the statistical overview, see also the table in Extended Data Figure 1-1.

All data were plotted with GraphPad Prism 7, and represented in bar plots as mean ± SEM. First, the data were tested for normality with D'Agostino-Pearson test. If they did not pass the parametric assumption, Kruskal–Wallis test followed by Dunn's test was performed for multiple comparison, and Mann–Whitney *U* test was applied for comparison of two unpaired datasets. In case the parametric assumption was passed, ordinary one-way ANOVA followed by Tukey's test for the multiple comparison, and Student's *t* test for two datasets were applied. The α level was set at 0.05. In the manuscript, the absolute values are shown as mean ± SEM, and *n* is the number of data points/number of cultures. The overview statistics including sample size, mean, SEM, *p* value, and result statistics for each figure are shown in Extended Data Figure 1-1 to Figure 7-1.

## Results

### RIM1/2 and Munc13-1 differentially contribute to SV distribution at the AZ

In order to investigate the expression levels of RIM1/2 and Munc13-1 in our experimental system, we generated RIM1/2 control and cDKO neurons by transducing continental hippocampal cultures from RIM1/2*^flox^* mice (Kaeser et al., 2011) with either inactivated Cre-recombinase (ΔCre) or Cre-recombinase (Cre), respectively. After 14 DIVs, we assessed protein expression of RIM1/2 and Munc13-1 in RIM1/2 control (ΔCre) and RIM1/2 cDKO (Cre) using Western blot analysis. We found that Cre expression in RIM1/2*^flox^* neurons leads to undetectable RIM1/2 levels, verifying successful removal of RIM1/2 proteins (Fig. 1*A*, left). Consistent with the previous reports (Deng et al., 2011), loss of RIM1/2 also resulted in severely reduced Munc13-1 protein levels (Fig. 1*A*, right), confirming the necessity of RIM in recruiting and stabilizing Munc13-1 at the AZ (Andrews-Zwilling et al., 2006).

10.1523/JNEUROSCI.1922-20.2020.f1-1 Figure 1-1 Values and statistics corresponding to Figure 1. Download Figure 1-1, DOCX file.

**Figure 2.**
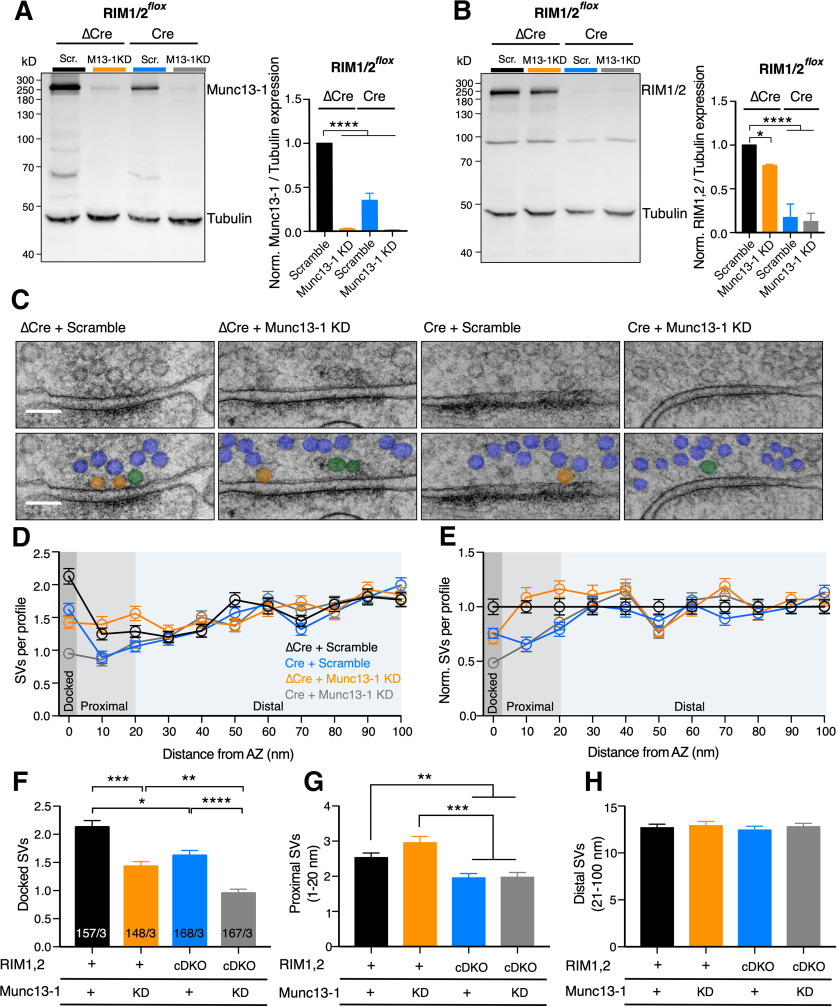
Munc13-1-independent impact of RIM on SV localization. ***A***, ***B***, Immunoblots (left) and bar plots (right) represent the expression of Munc13-1 (***A***) and RIM1/2 (***B***) in RIM1/2*^flox^* cultures infected with ΔCre + Scramble (Scr.) shRNA (black), ΔCre + 40 × 10^5^ infectious units (IU) Munc13-1 KD shRNA (orange), Cre + Scramble shRNA (blue), and Cre + 40 × 10^5^ Munc13-1 shRNA (gray). Tubulin expression was used as a loading control. Molecular weight markers are indicated on blots. The analysis was performed from three independent cultures. Significance was calculated using one-way ANOVA followed by Tukey's *post hoc* test (***A***: *F*_(3,8)_ = 133.8, *p* < 0.0001; ***B***: *F*_(3,8)_ = 48.07, *p* < 0.0001). ***C***, Example TEM images of RIM1/2*^flox^* hippocampal cultures infected as in ***A*** and ***B***. Top, Raw images. Bottom, Vesicles are color-coded according to their distance to the AZ membrane: docked SVs (orange), proximal SVs (green), distal SVs (blue). Scale bar, 100 nm. Additional example pictures are represented in Extended Data Figure 2-2. ***D***, ***E***, Plot represents the SV number as a function of distance from the AZ membrane (binned 10 nm; ***D***) and the same values normalized to the control (binned to 10 nm; ***E***). ***F–H***, Bar plots displaying the mean number of docked SVs (***F***), proximal SVs (1-20 nm) (***G***), and distal SVs (21-100 nm) (***H***). Bar graph label “+” indicates endogenous expression of RIM1/2 or Munc13-1. “KD” refers to Munc13-1 KD. “cDKO” refers to RIM1/2-deficient neurons. Number of synaptic profiles for ***D***–***H*** are indicated in ***F***. Values indicate mean ± SEM. *p ≤ 0.05; **p ≤ 0.01; ***p ≤ 0.001; ****p ≤ 0.0001; nonparametric one-way ANOVA with Kruskal–Wallis test followed by Dunn's *post hoc* test. For the statistical overview, see also the table in Extended Data Figure 2-1.

10.1523/JNEUROSCI.1922-20.2020.f1-2 Figure 1-2 Exemplary images corresponding to Figure 1. The figure shows raw micrographs (left) and the corresponding micrographs with marked vesicles (right). Vesicles are designated according to their distance to the AZ membrane: docked SVs (orange), proximal SVs (green), distal SVs (blue). Scale bar, 100 nm. Download Figure 1-2, TIF file.

To identify the specific effects of RIM1/2 and Munc13-1 on synapse ultrastructure, we cryo-preserved cultures derived from *Munc13-1*^−/−^ and *Munc13-1*^+/+^ neurons and RIM1/2 cDKO and control neurons under high pressure. We imaged 40 nm sections of synaptic profiles at high magnification in transmission electron microscopy (TEM) (Fig. 1*C*) and analyzed standard ultrastructure parameters, including PSD length, number of SVs docked at the AZ membrane (defined as the membrane opposite to the PSD), and the shortest distance of undocked vesicles to the AZ membrane (Fig. 1*B*). The AZ length was not different between the KO groups and their controls (Fig. 1*F*,*G*; RIM1/2 ΔCre: 281.8 ± 12.8 nm, *n* = 153/3; RIM1/2 Cre: 286.7 ± 8.95 nm, *n* = 161/3, *p* = 0.094; Munc13-1 WT: 312.3 ± 10.72 nm, *n* = 148/3; Munc13-1 KO: 333.4 ± 12.03 nm, *n* = 173/3, *p* = 0.382, Mann–Whitney test). RIM1/2 cDKO presynaptic terminals showed a 30% reduction in docked SVs per AZ compared with controls (Fig. 1*H*; RIM1/2 ΔCre: 1.10 ± 0.09, *n* = 153/3; RIM1/2 Cre: 0.73 ± 0.07, *n* = 161/3, *p* = 0.002, Mann–Whitney test). On the other hand, Munc13-1 KO synapses displayed a ∼70% reduction in SV docking (Fig. 1*I*; Munc13-1 WT: 1.57 ± 0.10, *n* = 148/3; Munc13-1 KO: 0.56 ± 0.07, *n* = 173/3, *p* < 0.0001, Mann–Whitney test). Thus, high-pressure freezing and TEM experiments revealed SV docking deficits in cultures lacking either Munc13-1 or RIM1/2, albeit to a different extent.

Earlier studies have proposed a role for RIM in tethering SVs close to the AZ membrane (Fernández-Busnadiego et al., 2013). While we cannot assess tethers in our high-pressure frozen samples, we investigated the respective roles of RIM1/2 and Munc13-1 in the localization of nondocked SVs within 100 nm of the AZ membrane. RIM1/2 cDKO presynaptic terminals displayed a reduced number of SVs within the first 20 nm of the AZ membrane (Fig. 1*D*,*J*; RIM1/2 ΔCre: 1.80 ± 0.12, *n* = 153/3; RIM1/2 Cre: 1.28 ± 0.10, *n* = 161/3, *p* = 0.0004, Mann–Whitney test), but no change in density of SVs from >20 nm up to 100 nm from the AZ membrane (Fig. 1*D*,*L*; RIM1/2 ΔCre: 8.96 ± 0.35, *n* = 153/3; RIM1/2 Cre: 8.60 ± 0.3, *n* = 161/3, *p* = 0.675, Mann–Whitney test). Therefore, to facilitate the discussion about the relative SV localization, we defined 20 nm as a border to separate “proximal” SVs (1-20 nm) from the “distal” SVs (21-100 nm) to the AZ membrane (Fig. 1*B*).

On the other hand, Munc13-1-deficient presynaptic terminals showed no change in AZ-proximal SV number because of a significant SV accumulation near the AZ membrane (Fig. 1*E*,*K*; Munc13-1 WT: 2.03 ± 0.12, *n* = 148/3; Munc13-1 KO: 2.09 ± 0.13, *n* = 173/3, *p* = 0.853, Mann–Whitney test). The different phenotype of two KOs in localization of SVs at the AZ-proximal region relative to their controls proposes that RIM localizes vesicles in the proximity to the AZ. Similar to RIM1/2 cDKO synapses, Munc13-1 KO synapses showed no change in distal SVs (Fig. 1*E*,*M*; Munc13-1 WT: 9.61 ± 0.34, *n* = 148/3; Munc13-1 KO: 10.23 ± 0.38, *p* = 0.677, *n* = 173/3, Mann–Whitney test). Therefore, neither RIM1/2 nor Munc13-1 is specifically involved in distal SV localization.

### RIM1/2 localizes SVs in the AZ proximity independent of Munc13-1

Since RIM1/2 deletion also results in a partial reduction of Munc13-1 protein levels (Deng et al., 2011), we aimed to separate RIM1/2 and Munc13-1 function by further reducing Munc13-1 protein in the RIM1/2 cDKO and control neurons using an shRNA knockdown (KD) approach (Fig. 2). Western blot analysis revealed a nearly complete abolishment of Munc13-1 protein in neuronal lysates infected with the shRNA (Fig. 2*A*). As expected, RIM1/2 proteins were diminished by ∼90% in RIM1/2 cDKO lysates (Fig. 2*B*). We also noted that KD of Munc13-1 in control neurons displayed a small but detectable reduction in RIM1/2 protein levels (Fig. 2*B*).

10.1523/JNEUROSCI.1922-20.2020.f2-1 Figure 2-1 Values and statistics corresponding to Figure 2. Download Figure 2-1, DOCX file.

10.1523/JNEUROSCI.1922-20.2020.f2-2 Figure 2-2 Exemplary images corresponding to Figure 2. The figure shows raw micrographs (left) and the corresponding micrographs with marked vesicles (right). Vesicles are designated according to their distance to the AZ membrane: docked SVs (orange), proximal SVs (green), distal SVs (blue). Scale bar, 100 nm. Download Figure 2-2, TIF file.

The KD of Munc13-1 in control neurons resulted in a decreased number of docked SVs compared with its control (Fig. 2*F*; RIM1/2 ΔCre + Scramble: 2.13 ± 0.11, *n* = 157/3; RIM1/2 ΔCre + Munc13-1 KD: 1.42 ± 0.09, *n* = 148/3, *p* = 0.0002, Kruskal–Wallis test). While SV docking was reduced by 30% in RIM1/2 cDKO synapses, the additional KD of Munc13-1 in RIM1/2 cDKO caused a more severe docking impairment (Fig. 2*F*; RIM1/2 Cre + Scramble: 1.61 ± 0.1, *n* = 168/3; RIM1/2 Cre + Munc13-1 KD: 0.95 ± 0.07, *n* = 167/3, *p* < 0.0001, Kruskal–Wallis test). Hence, both RIM and Munc13-1 play a substantial role in SV docking. Moreover, while the total number of SVs within AZ-proximal region of Munc13-1 KD in control neurons did not alter (Fig. 2*D*,*E*,G), it decreased in RIM1/2 cDKO synapses compared with the control (Fig. 2*D*,*E*,*G*; RIM1/2 ΔCre + Scramble: 2.53 ± 0.13, *n* = 157/3; RIM1/2 ΔCre + Munc13-1 KD: 2.95 ± 0.18, *n* = 148/3, *p* > 0.999; RIM1/2 Cre + Scramble: 1.95 ± 0.13, *n* = 168/3, *p* = 0.002, Kruskal–Wallis test). Interestingly, Munc13-1 KD had no further effect on AZ-proximal SV number in RIM1/2 cDKO synapses (Fig. 2*D*,*E*,*G*; RIM1/2 Cre + Scramble: 1.95 ± 0.13, *n* = 168/3, RIM1/2 Cre + Munc13-1 KD: 1.98 ± 0.14, *n* = 167/3, *p* > 0.999, Kruskal–Wallis test), illustrating a Munc13-1-independent role of RIM in positioning SVs within close proximity to the AZ. In addition, the SV number in the AZ-distal region was not affected by the loss of RIM1/2, Munc13-1, or both (Fig. 2*D*,*E*,*H*; Kruskal–Wallis (H) = 1.92, *p* = 0.587). Overall, our data demonstrate that RIM localizes SVs in proximity to the AZ in a Munc13-1-independent manner, while both RIM1/2 and Munc13-1 are required for SV docking.

### Deletion of RIM1/2 severely impairs SV priming and neurotransmitter release in glutamatergic hippocampal autaptic neurons

**Figure 3.**
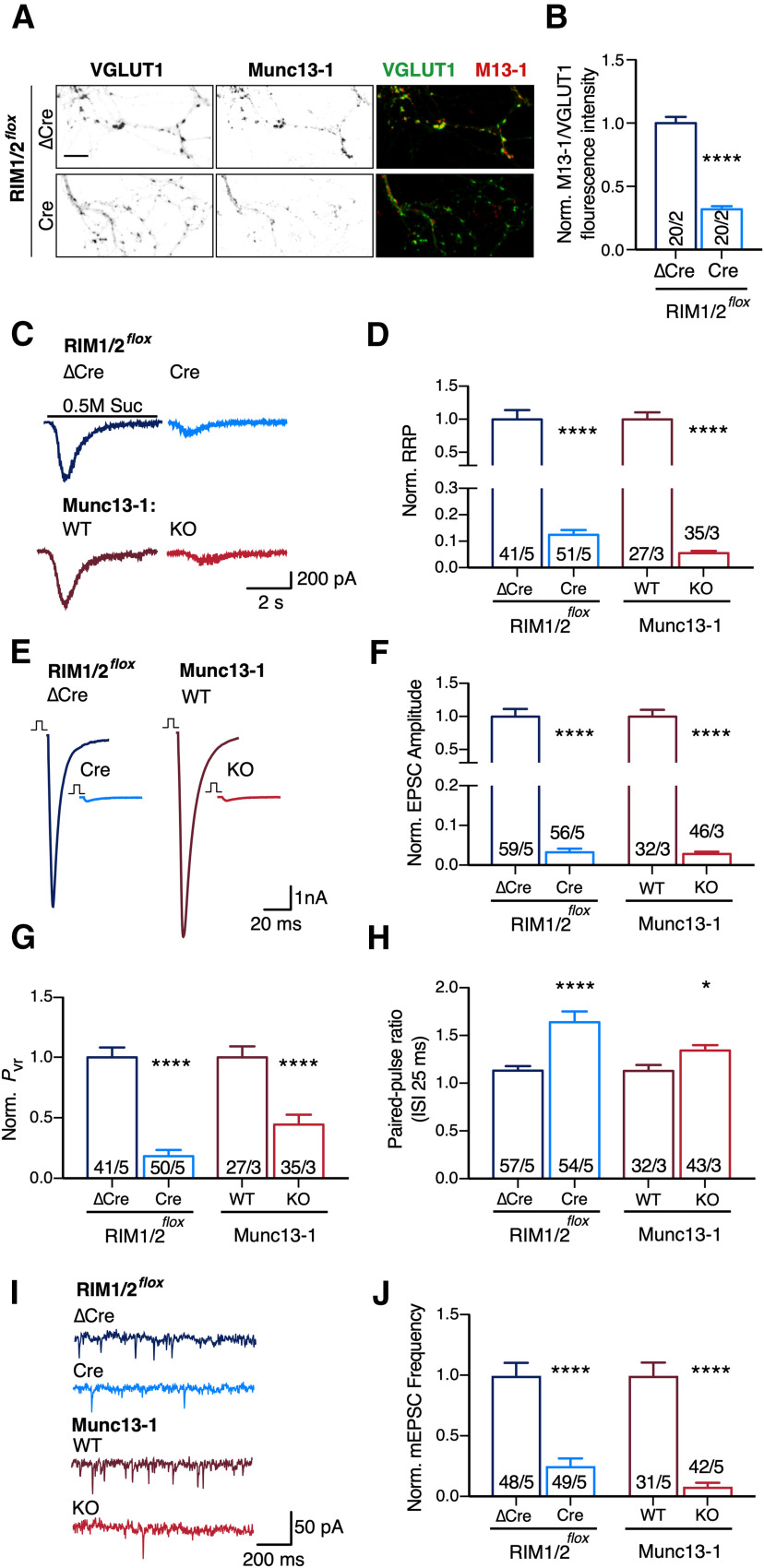
Comparison of synaptic properties of murine RIM1/2- and Munc13-1 KOs in autaptic hippocampal neurons. ***A***, Representative confocal microscopy projections display the immunofluorescence for Munc13-1 and VGLUT1 in RIM1/2 control and cDKO autaptic neurons. Scale bar, 10 µm. ***B***, Bar plot represents the ratiometric fluorescence intensity levels of Munc13-1 to VGLUT1 normalized to the control. Significance was calculated using Student's *t* test (*t*_(38)_ = 9.25, *p* < 0.0001). ***C***, Example traces of current induced by application of hypertonic solution (0.5 m sucrose [Suc], 5 s) to estimate the size of RRP in neurons derived from RIM1/2 control (navy blue), RIM1/2 cDKO (light blue), Munc13-1 WT (dark red), and Munc13-1 KO (light red). ***D***, Bar plots represent the normalized mean of RRP size (sucrose charge transfer). ***E***, Example traces of EPSCs from the same experimental groups as in ***C***. ***F***, Bar plot represents the normalized mean of EPSC amplitude. ***G***, Bar plot represents the normalized vesicular release probability calculated by dividing the charge of EPSC to the sucrose charge. ***H***, Bar plot represents the paired-pulse ratio (with an interstimulus interval of 25 ms). ***I***, Example traces of spontaneous release events from the same experimental groups as in ***C***. ***J***, Bar plot represents the normalized mean frequency of mEPSCs. All of the bar plots are normalized to the corresponding controls, except the paired-pulse ratio. Significances were calculated between the RIM1/2 control and cDKO (navy blue and light blue), and between Munc13-1 WT and KO (dark red and light red) using nonparametric *t* tests, followed by a Mann–Whitney test. All numbers in bars indicate the cell number/culture number. Data indicate normalized mean ± SEM. *p ≤ 0.05. ****p ≤ 0.0001. For the absolute values and statistical overview, see also the table in Extended Data Figure 3-1.

Based on our findings that RIM1/2 influences SV docking and SV localization near the AZ membrane, we asked how RIM1/2 and Munc13-1 contribute to SV priming and neurotransmitter release in RIM1/2 or Munc13-1-deficient glutamatergic autaptic neurons. To examine how loss of RIM1/2 affects Munc13-1 protein levels at the synapse, we performed quantitative immunocytochemistry. By normalizing Munc13-1 immunofluorescence intensity to the corresponding signals from the SV marker VGLUT1 (Camacho et al., 2017), we found that Munc13-1 levels were reduced by ∼70% in the presynaptic terminals of autaptic RIM1/2 cDKO neurons (Fig. 3*B*; *t*_(38)_ = 9.25, *p* < 0.0001, Student's *t* test). This supports the notion that RIM1/2 stabilizes Munc13-1 at the AZ (Andrews-Zwilling et al., 2006).

10.1523/JNEUROSCI.1922-20.2020.f3-1 Figure 3-1 Absolute values and statistics corresponding to Figure 3. Download Figure 3-1, DOCX file.

To compare the roles of RIM and Munc13-1 in SV priming activity, we estimated the size of the RRP of SVs. RRP of RIM1/2 cDKO or Munc13-1 KO autaptic neurons and their respective controls was estimated by measuring the transient postsynaptic charge component evoked by the pulsed application of hypertonic sucrose (Rosenmund and Stevens, 1996) (Fig. 3*C*). Compared with the control neurons, RRP size was severely reduced by Munc13-1 deletion (∼95%) or RIM1/2 deletion (∼88%) (Fig. 3*D*; RIM1/2 ΔCre: 0.7 ± 0.1 nC, *n* = 41/5; RIM1/2 Cre: 0.08 ± 0.01 nC, *n* = 51/5, *p* < 0.0001; Munc13-1 WT: 0.69 ± 0.09 nC, *n* = 27/3; Munc13-1 KO: 0.04 ± 0.006 nC, *n* = 35/3, *p* < 0.0001, Mann–Whitney test). Thus, the presence of RIM1/2 and Munc13-1 is required for efficient SV priming to occur, which goes in line with the SV docking role of RIM1/2 and Munc13-1 (Fig. 1). However, the impairment of SV priming was more drastic than the impairment in SV docking in both KOs.

To address the impact of RIM1/2 on Ca^2+^-evoked release in autaptic neurons, we analyzed the AP-evoked EPSCs (Fig. 3*E*). The EPSC amplitude in RIM1/2 cDKO autaptic neurons was reduced by ∼97%, similar to the loss of EPSC amplitude observed in Munc13-1 KO neurons (Fig. 3*F*; RIM1/2 ΔCre: 6.3 ± 0.72 nA, *n* = 59/5; RIM1/2 Cre: 0.18 ± 0.05 nA, *n* = 56/5, *p* < 0.0001; Munc13-1 WT: 7.1 ± 0.89 nA, *n* = 32/3; Munc13-1 KO: 0.19 ± 0.03 nA, *n* = 46/3, *p* < 0.0001, Mann–Whitney test). While a large portion of the EPSC amplitude decrease in the absence of RIM1/2 is likely attributable to reduced SV priming activity, it is known that RIM1/2 also regulates Ca^2+^ channel recruitment (Han et al., 2011; Kaeser et al., 2011). We thus predicted that RIM1/2 cDKO autaptic neurons also had less efficacious Ca^2+^ secretion-coupling than Munc13-1 KO neurons, affecting vesicular release probability (*P*_vr_). We computed the *P*_vr_ by calculating the ratio of the EPSC charge to the sucrose-induced charge. *P*_vr_ was decreased by ∼82% for RIM1/2 cDKO, while the loss of Munc13-1 led to a reduction of *P*_vr_ by only ∼56% (Fig. 3*G*; RIM1/2 ΔCre: 6.4 ± 0.56%, *n* = 41/5; RIM1/2 Cre: 1.11 ± 0.3%, *n* = 50/5, *p* < 0.0001; Munc13-1 WT: 8.08 ± 0.74%, *n* = 27/3; Munc13-1 KO: 3.50 ± 0.63%, *n* = 35/3, *p* < 0.0001, Mann–Whitney test). We independently probed the efficiency of release by recording postsynaptic responses to paired-pulse stimulation protocols. Consistent with our observations of *P*_vr_ changes, we noticed a strong facilitation in the RIM1/2-deficient neurons (Fig. 3*H*), which confirms the stark decrease in release probability. Munc13-1-deficient neurons, in line with the less drastic *P*_vr_ loss, showed a moderate increase in facilitation (Fig. 3*H*; RIM1/2 ΔCre: 1.13 ± 0.05, *n* = 57/5; RIM1/2 Cre: 1.63 ± 0.11, *n* = 54/5, *p* < 0.0001; Munc13-1 WT: 1.13 ± 0.06, *n* = 32/3; Munc13-1 KO: 1.34 ± 0.06, *n* = 43/3, *p* = 0.01, Mann–Whitney test).

We also examined the impact of RIM1/2 and Munc13-1 on spontaneous release by analyzing the frequency of mEPSCs (Fig. 3*I*). Consistent with the reduced priming function, loss of both RIM and Munc13-1 proteins strongly impaired mEPSC frequency (Fig. 3*J*; RIM1/2 ΔCre: 6.67 ± 0.72 Hz, *n* = 48/5; RIM1/2 Cre: 1.48 ± 0.33 Hz, *n* = 49/5, *p* < 0.0001; Munc13-1 WT: 8.62 ± 0.92 Hz, *n* = 31/3; Munc13-1 KO: 0.72 ± 0.23 Hz, *n* = 42/3, *p* < 0.0001, Mann–Whitney test).

These results confirm that both RIM1/2 and Munc13-1 are required for neurotransmission. While our data verify previous findings that both RIM1/2 and Munc13-1 regulate SV priming and release efficiency, we extend these findings by demonstrating the relative contributions of RIM1/2 and Munc13-1 to each of these processes. We found that RIM plays a major role in determining vesicular release probability and Munc13 predominantly controls vesicle priming.

### RIM1/2 cDKO loss of function does not depend on Munc13-1 concentrations

**Figure 4.**
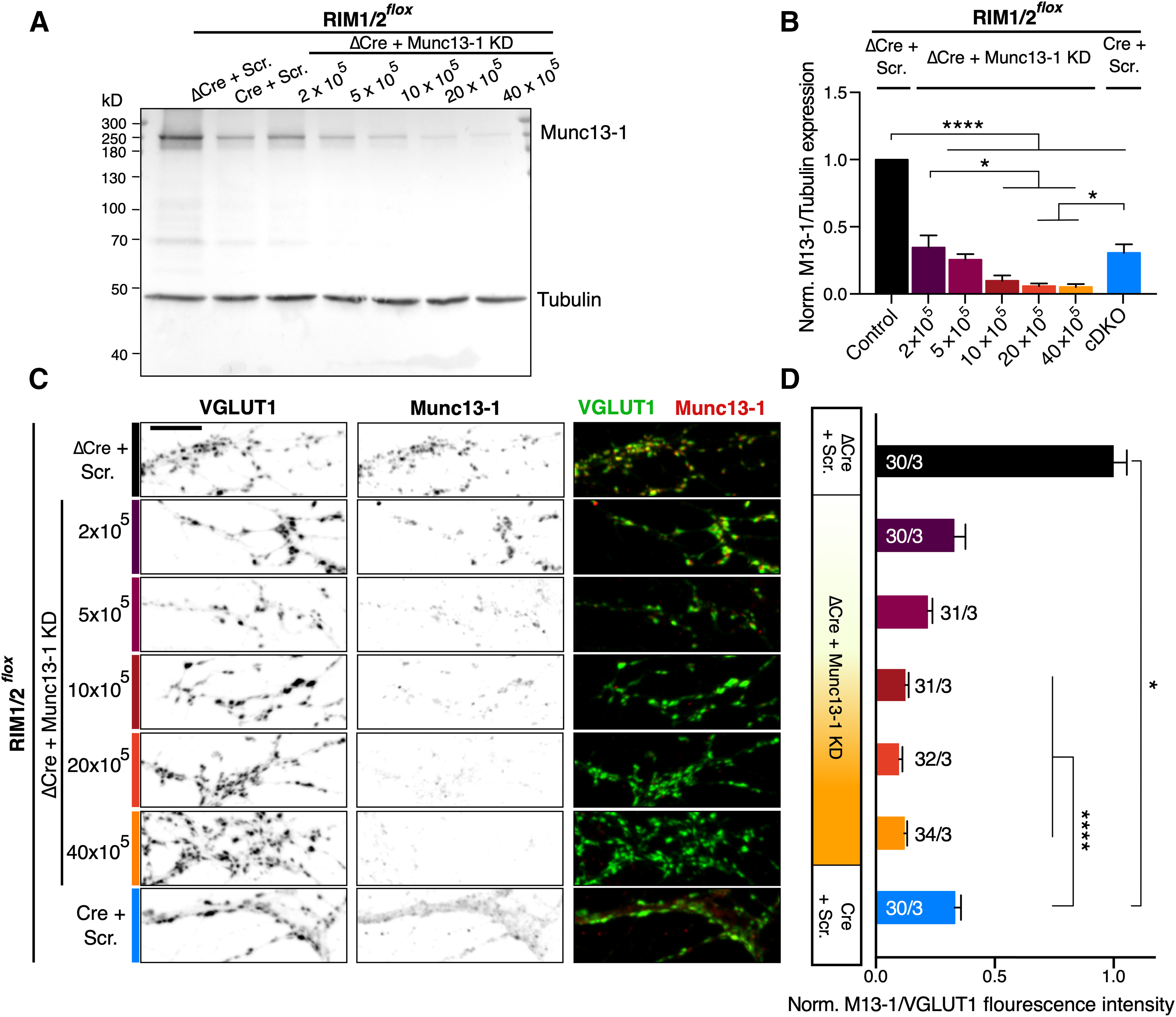
Munc13-1 titration in hippocampal neurons. ***A***, Immunoblot detecting Munc13-1 expression in RIM1/2*^flox^* lysates from cultured hippocampal neurons infected with ΔCre + Scramble (Scr.) shRNA as control, ΔCre + Munc13-1 KD shRNAs (2-40 × 10^5^ IU) to produce Munc13-1 dose gradients, and Cre recombinase + Scramble shRNA to create RIM1/2 cDKO. Tubulin expression was used as a loading control. Left, Molecular weight markers. ***B***, Bar plot represents ratiometric quantification of Munc13-1 expression levels to tubulin. Data were collected from three independent cultures and normalized to the corresponding control. Significances were calculated using one-way ANOVA followed by Tukey's *post hoc* test (*F*_(6,14)_ = 46.79, *p* < 0.0001). *p ≤ 0.05. ****p ≤ 0.0001. ***C***, Representative confocal microscopy projections display immunofluorescence intensity of Munc13-1 and VGLUT1 in the same experimental groups as in ***A***. Scale bar, 10 µm. ***D***, Horizontal bar plot represents the ratiometric fluorescence intensity levels of Munc13-1 to VGLUT1 normalized to the control. For each neuron, ∼50 synapses were measured and averaged. Statistical significances are represented only in comparison with RIM1/2 cDKO. Significances and *p* values were calculated with a nonparametric one-way ANOVA with Kruskal–Wallis test followed by Dunn's *post hoc* test (H = 128.2, *p* < 0.0001). *p ≤ 0.05. ****p ≤ 0.0001. Data indicate normalized mean ± SEM. For the statistical overview, see also the table in Extended Data Figure 4-1.

RIM influences Munc13-1 function by maintaining overall Munc13-1 protein levels in the synapse (Fig. 3*A*) and by activating Munc13-1 through disrupting the Munc13-1 homodimers (Deng et al., 2011; Camacho et al., 2017). To better understand the relative weight of these two functions of RIM and to gain a better mechanistic understanding of RIM's role in Munc13-1-mediated vesicle priming, we performed a graded shRNA-mediated KD of Munc13-1. To do so, cultures were infected with five doses of Munc13-1 shRNA ranging from 2 to 40 × 10^5^ infectious units (IU). KD efficiency was assessed by both immunoblotting the hippocampal lysates (Fig. 4*A*) and immunocytochemistry on autaptic hippocampal cultures (Fig. 4*C*). Both analyses confirmed that by elevating the concentration of shRNA, Munc13-1 protein level was reduced in a dose-dependent manner, ranging from 70% to 95% (Fig. 4*B*,*D*).

10.1523/JNEUROSCI.1922-20.2020.f4-1 Figure 4-1 Values and statistics corresponding to Figure 4. Download Figure 4-1, DOCX file.

**Figure 5.**
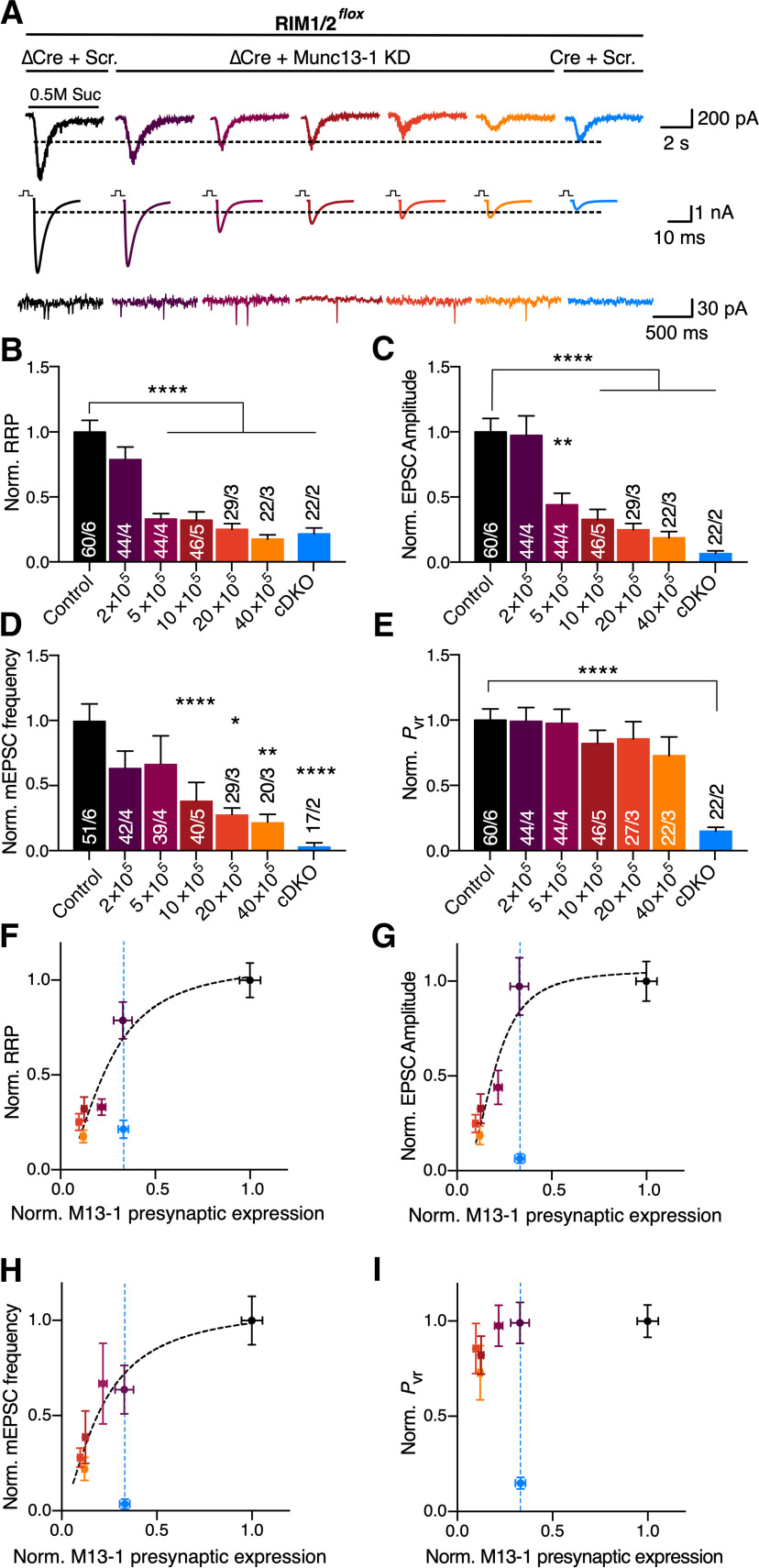
Quantification of RIM-dependent loss of Munc13-1 activity on synaptic properties. ***A***, Sample traces of current induced by hypertonic sucrose to estimate the RRP size (top), sample traces of EPSCs (middle), and mEPSCs (bottom). Autaptic hippocampal neurons were infected with ΔCre + Scramble (Scr.) shRNA as control, ΔCre + Munc13-1 KD shRNAs (2-40 × 10^5^ IU) to produce Munc13-1 dose gradients, and Cre recombinase + Scramble shRNA to create RIM1/2 cDKO. Dashed lines indicate the maximum current amplitude of RIM1/2 cDKO. ***B–E***, Bar plots represent the normalized mean RRP defined as a charge measured by hypertonic sucrose application (***B***), normalized EPSC amplitude (***C***), normalized mEPSC frequency (***D***), and normalized *P*_vr_ (***E***). ***F***–***I***, Plots represent the normalized RRP size (***F***), EPSC amplitude (***G***), mEPSC frequency (***H***), and *P*_vr_ (***I***) as a function of normalized Munc13-1/VGLUT1 presynaptic expression. ***F***-***I***, Black dashed lines indicate fitting with the Hill equation. Blue dashed line indicates Munc13-1/VGLUT1 expression in RIM1/2 cDKO neurons. Data indicate normalized mean ± SEM. *p ≤ 0.05; **p ≤ 0.01; ****p ≤ 0.0001; nonparametric one-way ANOVA with Kruskal–Wallis test followed by Dunn's *post hoc* test. For the absolute values and statistical overview, see also the table in Extended Data Figure 5-1.

We then proceeded by comparing RRP sizes between the groups and the control (Fig. 5*A*,*B*). Strikingly, despite similar Munc13-1 levels (30% of control) in both RIM1/2 cDKO and lowest dose of Munc13-1 shRNA, the 2 × 10^5^ IU Munc13-1 shRNA did not significantly alter the RRP size (Fig. 5*B*; RIM1/2 ΔCre + Scramble: 0.42 ± 0.05 nC, *n* = 60/6; RIM1/2 ΔCre + Munc13-1 shRNA 2 × 10^5^ IU: 0.40 ± 0.06, *n* = 44/4, *p* > 0.999, Kruskal–Wallis test). Only at higher doses of shRNA, when Munc13-1 protein levels were reduced by 80%-95%, the deficit in SV priming reached similar levels to RIM1/2 cDKO (Fig. 5*B*). Assuming that the role of RIM in priming is confined to Munc13-1 activation (Deng et al., 2011), our data indicate that RIM1/2 increases the effectiveness of Munc13-1 in SV priming by approximately fourfold.

10.1523/JNEUROSCI.1922-20.2020.f5-1 Figure 5-1 Absolute values and statistics corresponding to Figure 5. Download Figure 5-1, DOCX file.

When we compared the effect of graded Munc13-1 KD on evoked responses and spontaneous release events, we found that EPSC amplitudes and mEPSC frequency followed the reduction of RRP size (Fig. 5*A–D*). However, only with the KD of Munc13-1 protein level by >90%, the EPSC amplitude and mEPSC frequency reached the reduced level of the RIM1/2 cDKO neurons (Fig. 5*C*,*D*). Thus, RIM1/2 cDKO revealed the most severe phenotype in both parameters of EPSC amplitude and mEPSC frequency compared with the control.

In turn, *P*_vr_ was not majorly affected by Munc13-1 levels, whereas we observed a significant reduction in *P*_vr_ in RIM1/2 cDKO neurons (Fig. 5*E*). This suggests that reducing Munc13-1 levels does not impair Ca^2+^-secretion coupling, in contrast to the effect of eliminating RIM1/2.

By plotting SV priming and release as a function of Munc13-1 protein levels at the presynapse, we created dose–response plots of Munc13-1 for RRP size, EPSC amplitude, mEPSC frequency, and *P*_vr_ (Fig. 5*F–I*). We fitted the data from RRP size, EPSC amplitude, and mEPSC frequency measurements with a standard Hill equation (Arancillo et al., 2013) (see also Materials and Methods) to determine the relative sensitivity of synaptic function to Munc13-1 protein level and to define putative cooperativities. The fits show that the half-maximal SV priming, Ca^2+^-evoked release, and mEPSC frequency are achieved at 25%, 19%, and 20% of WT Munc13-1 level, respectively (Fig. 5*F–H*). These processes follow positive cooperativity functions (1.9 for SV priming, 2.6 for Ca^2+^-evoked release, and 1.6 for mEPSC frequency). The similar sensitivity of Munc13-1 levels on the SV priming and mEPSC frequency functions suggests that the RRP is likely the source of vesicles released during spontaneous miniature events. These data also suggest that, at concentrations <30% of WT levels, Munc13-1 is a rate-limiting factor for priming and release.

Additionally, we observed that the impairment in physiological measurements in RIM1/2 cDKO synapses (which express ∼30% of Munc13-1; Fig. 5*F–I*) did not follow the dose–response curves generated by Munc13-1 KD. This indicates that RIM1/2 cDKO loss of function does not only depend on Munc13-1 concentrations.

### RIM1/2 controls *P*_vr_ independent from Munc13-1 but regulates SV docking and priming together with Munc13-1

**Figure 6.**
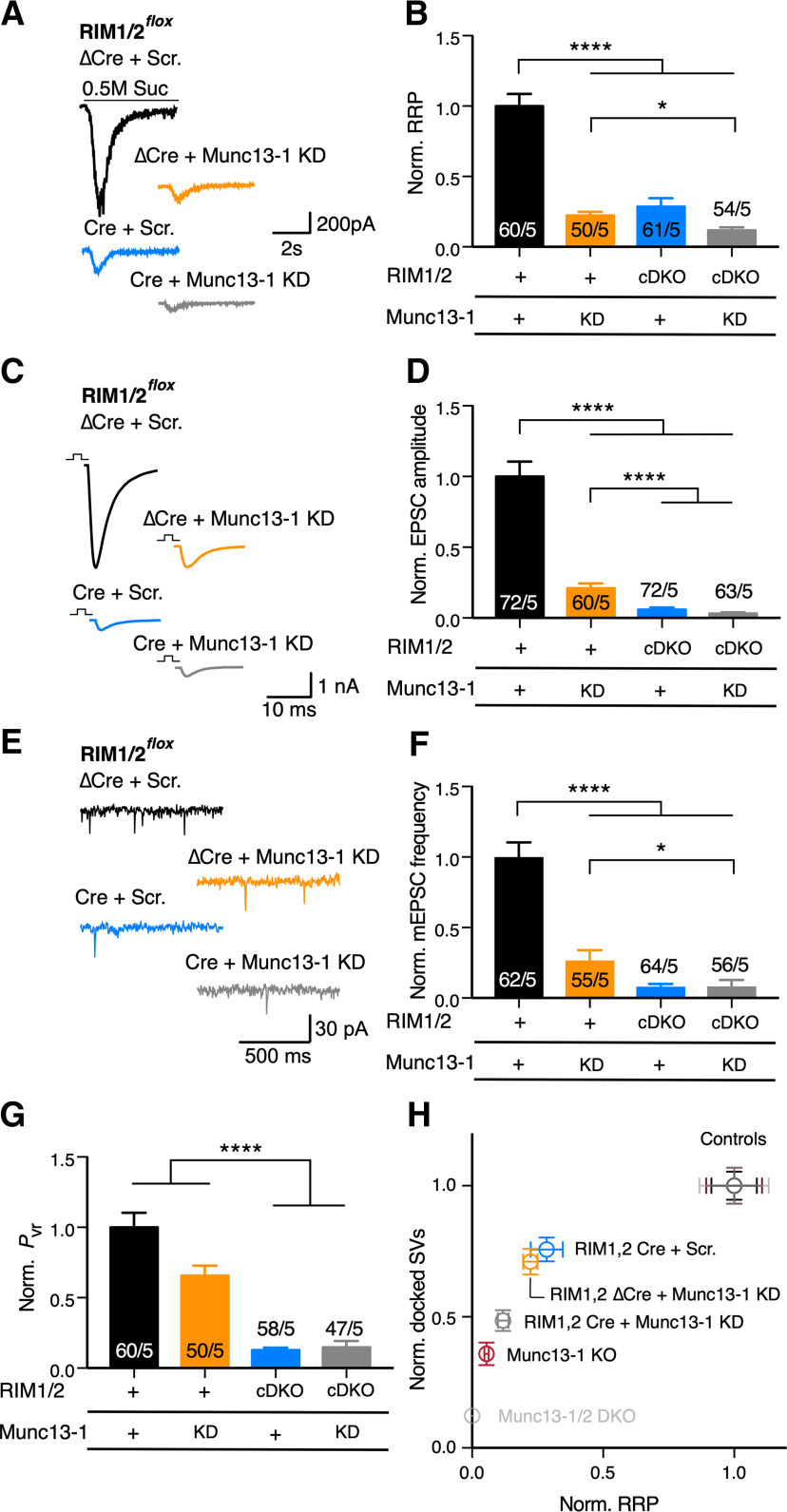
RIM-independent function of Munc13-1 on synaptic properties. ***A***, Sample traces represent the current evoked by hypertonic sucrose as an estimate for RRP in RIM1/2*^flox^* cultures infected with ΔCre + Scramble (Scr.) shRNA (black), ΔCre + 40 × 10^5^ IU of Munc13-1 KD shRNA (orange), Cre + Scramble shRNA (blue), and Cre + 40 × 10^5^ Munc13-1 shRNA (gray). ***B***, Bar plot represents the normalized mean of charge released by hypertonic sucrose (referred to as RRP). ***C***, ***D***, Sample traces of EPSC (***C***) and bar plot of normalized EPSC amplitude. ***E***, ***F***, Sample traces of spontaneous release events (***E***), and the bar plot represents the normalized mean mEPSC frequency (***F***). ***G***, Bar plot represents the normalized *P*_vr_. Normalization in all of the bar plots is performed relative to the corresponding control (ΔCre + scramble; black). In graph labels, “+” refers to the endogenous expression. “KD” refers to the Munc13-1 KD. “cDKO” refers to RIM1/2 deficiency. Numbers in bars indicate the cell number/culture number. Data indicate normalized mean ± SEM. *p ≤ 0.05; ****p ≤ 0.0001; nonparametric one-way ANOVA with Kruskal–Wallis test followed by Dunn's *post hoc* test. For the absolute values and statistical overview, see also the table in Extended Data Figure 6-1. ***H***, Plot represents the SV docking/priming relationship. Munc13-1/2 DKO data are adapted from Camacho et al. (2017). Controls represent ΔCre + Scr., Munc13-1 WT, and Munc13-1/2 DKO rescue with WT Munc13-1 (Camacho et al., 2017).

To further assess the role of RIM independent of Munc13-1 function, we treated RIM1/2 control or deficient neurons with the highest dose Munc13-1 shRNA (40 × 10^5^ IU). Near-complete removal of Munc13-1 in RIM1/2 cDKO neurons did not further reduce priming process and mEPSC frequency beyond the level of RIM1/2 cDKO alone (Fig. 6*B*; RIM1/2 Cre + Scramble: 0.12 ± 0.03 nC, *n* = 61/5; RIM1/2 Cre + Munc13-1 KD: 0.05 ± 0.008 nC, *n* = 54/5, *p* = 0.057; Fig. 6*F*; RIM1/2 Cre + Scramble: 0.50 ± 0.12 Hz, *n* = 64/5; RIM1/2 Cre + Munc13-1 KD: 0.50 ± 0.34 Hz, *n* = 56/5, *p* > 0.999, Kruskal–Wallis test), and showed a significant decrease compared with Munc13-1 KD in control neurons (Fig. 6*B*; RIM1/2 ΔCre + Munc13-1 KD: 0.08 ± 0.009 nC, *n* = 50/5; RIM1/2 Cre + Munc13-1 KD: 0.05 ± 0.008 nC, *n* = 54/5, *p* = 0.015; Fig. 6*F*; RIM1/2 ΔCre + Munc13-1 KD: 1.07 ± 0.22 Hz, *n* = 55/5; RIM1/2 Cre + Munc13-1 KD: 0.50 ± 0.34 Hz, *n* = 56/5, *p* = 0.026, Kruskal–Wallis test). These data provide supporting evidence for a function of RIM as an activator of Munc13-1 in SV priming (Deng et al., 2011; Camacho et al., 2017), and suggest that both RIM and Munc13-1 are required for priming function. Moreover, removal of Munc13-1 in the RIM1/2-deficient neurons did not further impair the EPSC amplitude (Fig. 6*D*; RIM1/2 Cre + Scramble: 0.23 ± 0.06 nA, *n* = 72/5; RIM1/2 Cre + Munc13-1 KD: 0.13 ± 0.04 nA, *n* = 63/5, *p* = 0.745, Kruskal–Wallis test) and *P*_vr_ (Fig. 6*G*; RIM1/2 Cre + Scramble: 0.8 ± 0.11%, *n* = 58/5; RIM1/2 Cre + Munc13-1 KD: 0.95 ± 0.29%, *n* = 47/5, *p* > 0.999, Kruskal–Wallis test), again emphasizing that RIM predominantly controls the Ca^2+^-triggered release and *P*_vr_.

10.1523/JNEUROSCI.1922-20.2020.f6-1 Figure 6-1 Absolute values and statistics corresponding to Figure 6. Download Figure 6-1, DOCX file.

Recent studies correlated SV docking to the priming process, where the loss of SV docking was accompanied by loss of SV priming (Siksou et al., 2009; Imig et al., 2014). As our molecular and genetic manipulations of RIM1/2 and Munc13-1 protein levels provided a range of SV docking and priming impairments, we used these data to obtain the levels of SV priming as a function of the SV docking activity. When plotting these two functions (Fig. 6*H*), we found that Munc13- and RIM-dependent docking and priming function did not show a linear correlation. Indeed, vesicle priming was more sensitive to protein levels than SV docking in all RIM and Munc13 KOs/KDs. This may indicate either a lower stoichiometry of RIM and Munc13 for SV docking than priming, or that the 2D ultrastructural image analysis has lower resolution for detecting an SV docking deficit. Furthermore, the effect on SV docking/priming observed in the absence of RIM illustrated a sensitivity defined by the reduction of Munc13-1 protein levels. This supports the notion that the role of RIM1/2 in SV docking and priming can be simply explained by its role in recruiting and activating Munc13-1, as previously proposed (Andrews-Zwilling et al., 2006; Deng et al., 2011; Camacho et al., 2017).

### RIM1/2 does not contribute to activity-dependent RRP augmentation

**Figure 7.**
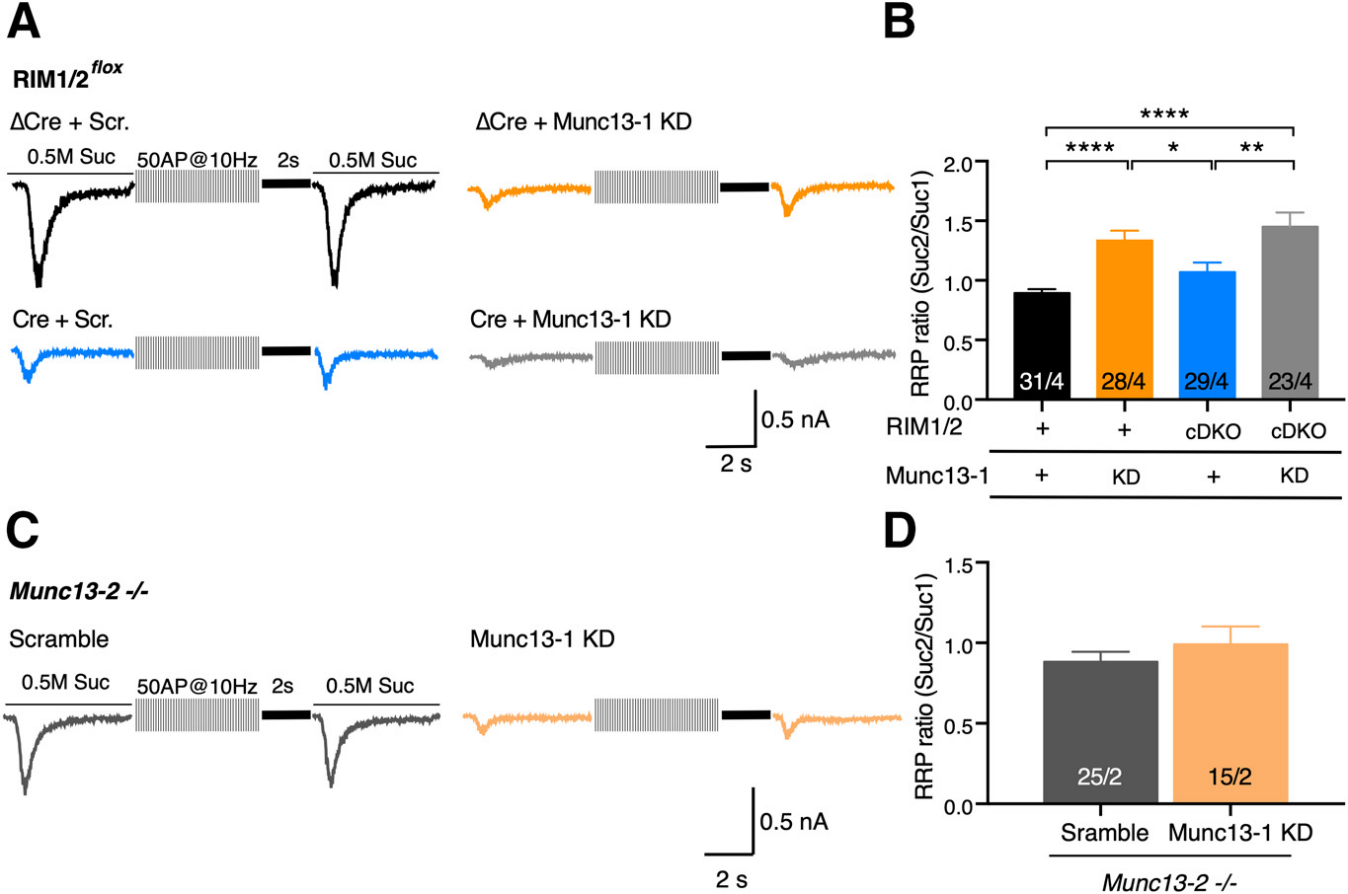
The regulation of RRP augmentation in the absence of RIM and Munc13-1. ***A***, Sample traces represent RRP augmentation protocol in RIM1/2*^flox^* cultures infected with ΔCre + Scramble (Scr.) shRNA (black), ΔCre + 40 × 10^5^ IU Munc13-1 KD shRNA (orange), Cre + Scramble shRNA (blue), and Cre + 40 × 10^5^ Munc13-1 shRNA (gray). After initial sucrose-evoked charge measurement (Suc1), 50 APs at 10 Hz were applied followed by another sucrose-evoked charge measurement 2 s after (Suc2). ***B***, Bar plot represents the mean RRP augmentation (ratio of Suc2 to Suc1) in neurons described in ***A***. Graph labels indicate endogenous expression “+.” “KD” refers to the Munc13-1 KD. “cDKO” refers to RIM1/2-deficient neurons; nonparametric one-way ANOVA with Kruskal–Wallis test followed by Dunn's *post hoc* test. ***C***, Sample traces of RRP augmentation protocol applied on Munc13-2 KO neurons with Munc13-1 KD (40 × 10^5^ shRNA IU). ***D***, Bar plot represents the measurement of RRP augmentations (Suc2/Suc1) in neurons as described in ***C***. The nonparametric *t* test, followed by Mann–Whitney test, did not show differences between Munc13-2 KO with Scramble and Munc13-1 KD. Numbers in bar plots indicate the cell number/culture number. Data indicate mean ± SEM. *p ≤ 0.05; **p ≤ 0.01; ****p ≤ 0.0001. For the statistical overview, see also the table in Extended Data Figure 7-1.

Our ultrastructural results suggested that RIM recruits SVs to the proximity of AZ membrane (Fig. 2); therefore, we investigated whether this function of RIM supplies SVs to the activity-dependent RRP augmentation that occurs in the absence of Munc13-1 (Rosenmund et al., 2002). To do so, we probed the RRP size by hypertonic sucrose application before and 2 s after 50 APs at 10 Hz high-frequency stimulation (Rosenmund et al., 2002) (Fig. 7*A*). Munc13-1 KD in either control or RIM1/2 cDKO synapse exhibited an activity-dependent augmentation of RRP size (RRP ratio: Fig. 7*B*; RIM1/2 ΔCre + Scramble: 0.89 ± 0.03, *n* = 31/4; RIM1/2 ΔCre + Munc13-1 KD: 1.33 ± 0.08, *n* = 28/4, *p* < 0.0001; RIM1/2 Cre + Munc13-1 KD: 1.45 ± 0.11, *n* = 23/4, *p* < 0.0001, Kruskal–Wallis test). This suggests that activity-dependent RRP augmentation occurs in the absence of RIM1/2, only when Munc13-1 levels are extremely low or completely abolished. Consistent with this, elimination of RIM1/2 alone, a condition in which Munc13-1 levels are reduced but not absent, did not result in high-frequency stimulation-mediated RRP augmentation (Fig. 7*B*; RIM1/2 ΔCre + Scramble: 0.89 ± 0.03, *n* = 31/4; RIM1/2 Cre + Scramble: 1.07 ± 0.08, *n* = 29/4, *p* = 0.854, Kruskal–Wallis test). Hence, RRP augmentation requires the absence of Munc13-1 but does not rely on the proximal localization of SVs to the AZ membrane by RIM1/2.

10.1523/JNEUROSCI.1922-20.2020.f7-1 Figure 7-1 Values and statistics corresponding to Figure 7. Download Figure 7-1, DOCX file.

Which molecule underlies activity-dependent RRP size augmentation in the absence of Munc13-1? In hippocampal neurons, Munc13-2 is expressed at low, but detectable, levels and is responsible for the remaining synaptic transmission in Munc13-1-deficient glutamatergic neurons (Rosenmund et al., 2002). The brain-specific isoform of Munc13-2 (bMunc13-2), in particular, has been postulated to participate in augmentation (Lipstein et al., 2012). To examine whether Munc13-2 is indeed responsible for RRP augmentation, we used Munc13-2-deficient neurons (Munc13-2 KO) and reduced Munc13-1 expression levels by shRNA-mediated KD (40 × 10^5^ IU) and examined activity-dependent RRP augmentation (Fig. 7*C*,*D*). We found that even with Munc13-1 KD, which revealed high-frequency stimulation-dependent RRP augmentation in control and RIM1/2 cDKO neurons, no RRP augmentation was observed in the absence of Munc13-2 (Fig. 7*D*; Scramble: 0.88 ± 0.06, *n* = 25/2; Munc13-1 KD: 0.99 ± 0.11, *n* = 15/2, *p* = 0.7, Mann–Whitney test). These data demonstrate that (1) Munc13-2 does indeed mediate RRP augmentation, (2) the absence of Munc13-1 is necessary to unmask RRP augmentation, and (3) RIM1/2 is not necessary for Munc13-2's priming activity or contribution to RRP augmentation.

## Discussion

Many proteins in the AZ work in concert to efficiently transduce presynaptic APs into neurotransmitter release. To understand how the AZ mediates presynaptic function, it is crucial to consider individual proteins in conjunction with their interaction partners. Here, we explored the extent to which RIM individually plays direct roles in SV localization and neurotransmission, versus its secondary roles through interactions with Munc13-1. We find that loss of RIM1/2 causes two effects on ultrastructure: impaired AZ-proximal localization of SVs and reduced SV docking. The docking deficit, in the absence of RIM, likely stems from a lack of activation and stabilization of Munc13-1. However, reduction of AZ-proximal SVs, which did not occur in Munc13-1 KO, reflects RIM's independent role in SV localization. Similar to the RIM's docking function, electrophysiological analysis showed that priming function of RIM depends on Munc13-1. However, the role of RIM in SV localization does not affect activity-dependent RRP augmentation. This work provides a finer view of the extent to which RIM and Munc13 cooperate to achieve SV recruitment, docking, priming and fusion.

### Role of RIM and Munc13-1 in SV localization

Recent advances in cryofixation techniques of neuronal tissue have emphasized the connection between synaptic ultrastructure and function, such as revealing the morphologic equivalent of SV priming as SV docking using high-pressure freezing fixation (Siksou et al., 2009; Imig et al., 2014). Therefore, we examined SV docking and distribution in high-pressure frozen synapses of RIM and/or Munc13-1-deficient synapses and achieved two important findings:

First, we show that RIM influences vesicle docking, which is consistent with the previous reports in murine synapses (Han et al., 2011; Kaeser et al., 2011; S. S. Wang et al., 2016) and in *Caenorhabditis elegans* (Gracheva et al., 2008). Nevertheless, the effect of RIM on SV docking is only attributable to Munc13-1 activation. This largely confirms the dogma of RIM acting upstream of Munc13-1 in SV docking (Han et al., 2011; Camacho et al., 2017).

Second, we find that RIM influences the distribution of SVs near the plasma membrane. This is different from Munc13-1 KO (Fig. 1) and Munc13-1/2-deficient synapses (Siksou et al., 2009; Imig et al., 2014), which demonstrated an enriched density of SVs near to the plasma membrane, but revealed severe docking deficits. The accumulation of SVs at AZ-proximal regions, which disappears with loss of RIM, demonstrates that the AZ-proximal SV localization relies on RIM, independent of Munc13-1. This finding extends the observations from cryo-electron tomographic studies in synaptosomes, where SVs connected with short protein “tethers” to the AZ membrane depend partially on RIM1α (Fernández-Busnadiego et al., 2010, 2013). We believe that these findings are not affected by synapse size, as previous studies reported that RIM1/2 cDKO does not impair total SV number, PSD length, or bouton size (Kaeser et al., 2011; Acuna et al., 2016) and Munc13-1/2 DKO does not affect SV number per terminal and PSD length (Imig et al., 2014). In neuromuscular junction synapses of *C. elegans*, RIM homolog, Unc10, deficiency also impairs SV localization to the dense projections (Weimer et al., 2006), suggesting an evolutionarily conserved role for RIM in SV localization. While SV localization near the AZ membrane is mediated by RIM, other AZ proteins, such as Piccolo, Bassoon, Liprin-α3, and Liprin-α2, influence the long-range SV distribution by modulating early stages of AZ assembly and vesicle formation (Mukherjee et al., 2010; Spangler et al., 2013; Wong et al., 2018; Ackermann et al., 2019). Thus, AZ proteins act in a hierarchical order by determining the precise SV localization.

What is the physiological relevance of vesicle tethering? One hypothesis is that tethering brings SVs in close proximity to the plasma membrane to make SVs readily available for entrance into the RRP (Hallermann and Silver, 2013). However, when we examined activity-dependent changes in RRP size, a condition that shows RRP augmentation in Munc13-1-deficient synapses (Rosenmund et al., 2002), we find that RIM does not affect this phenomenon. This argues that the physiological role of RIM in tethering function manifests by different means. Essentially, the function of tethers depends on their molecular composition and interactions with scaffolds. Bassoon, a protein that modulates long-range vesicle localization, tethers SVs by reloading SVs into the pool (Hallermann et al., 2010). Nevertheless, the fact that RIM-Rab3 complex anchors SVs close to both Ca^2+^ channels and plasma membrane (Han et al., 2011; Kaeser et al., 2011; de Jong et al., 2018) may indicate that RIM's tethering function is a product of several molecular interactions. Therefore, the functional role of RIM in SV localization may be resolved by other electrophysiological measurements as well as by providing identity to the composition of tethers at the AZ.

### Role of RIM and Munc13 isoforms in vesicular release probability

RIM defines the efficiency of release by recruiting Ca^2+^ channels (Han et al., 2011; Kaeser et al., 2011), binding to PIP2 rich membranes (de Jong et al., 2018) and interacting with Rab3 (Y. Wang et al., 1997; X. Wang et al., 2001; Fukuda, 2003; Schluter et al., 2006). In addition, in *C. elegans*, the interaction of Unc10 with Unc13 modulates *P*_vr_ (Zhou et al., 2013; Liu et al., 2019). However, we show that, in mammals, the effect of RIM on *P*_vr_ is independent from Munc13-1. Then what causes loss of *P*_vr_ in Munc13-1 KO neurons? While previous work has demonstrated that Munc13 KD impairs Ca^2+^ entry in hippocampal mass culture (Calloway et al., 2015), recent work in Munc13-1/2 DKO autaptic neurons shows no effect on presynaptic Ca^2+^ signal (Brockmann et al., 2020). As our data show that Munc13-1 reduction does not have a major effect on vesicular release probability, our findings are consistent with a lack of Munc13-1 effect on Ca^2+^ influx. Since only the total absence of Munc13-1, but not varying Munc13-1 levels, impairs *P*_vr_, we presume that priming with the alternative Munc13 isoform, Munc13-2, results in low release efficiency SVs. The function of Munc13-2 is only unmasked in the complete absence of Munc13-1, and not even revealed in the case of drastically reduced Munc13-1 expression levels. Furthermore, while only Munc13-2 is responsible for activity-dependent augmentation of RRP in a RIM- and Munc13-1-independent manner, both Munc13-1 and Munc13-2 contribute to dynamic changes in release probability through short-term plasticity by lowering the energy barrier for vesicle fusion (Rosenmund et al., 2002; Basu et al., 2007). Molecularly, these functions take place by activation of regulatory domains that bind to CaM, DAG (via C1 domain), and Ca^2+^ (via C2B domain) (Rhee et al., 2002; Junge et al., 2004; Shin et al., 2010; Lipstein et al., 2013).

### Other pathways of docking/priming parallel to RIM-Munc13-1

While the relevance of RIM-Munc13-1 interactions in SV docking and release at small central synapse is clear, alternative paths exist that allow SVs to become fusion-competent. For example, bMunc13-2 or Munc13-3 does not require RIM and is differentially expressed in specific brain regions (Augustin et al., 1999b). Our ultrastructural and physiological data from Munc13-1 KD in RIM1/2 cDKO neurons, as well as Munc13-1 KO neurons, suggest that bMunc13-2, which does not require activation via RIM, accounts for ∼30% of docking and ∼5% of priming. Moreover, in invertebrate synapses, long and short Munc13s exist that form distinct complexes with AZ proteins and regulate different forms of release similar to Munc13-1, -2, and -3 (Brose et al., 1995; Aravamudan et al., 1999; Richmond et al., 1999; Kohn et al., 2000; Rosenmund et al., 2002; Bohme et al., 2016; Liu et al., 2019). An interesting observation from our study is that RIM1/2 cDKO shows more severe impact on SV priming in glutamatergic autaptic neurons compared with GABAergic synapses in hippocampal mass culture (Deng et al., 2011). Although these differences could be attributed to the experimental systems, it is known that GABAergic neurons, in contrast to glutamatergic neurons, redundantly use Munc13-2 for priming (Augustin et al., 1999a; Varoqueaux et al., 2002). The synapse-type-specific AZ protein composition is also evident in other vertebrate synapses. For example, in hippocampus mossy fiber synapses, RIM expression is low, and Munc13-1 uses RIM-BP to prime SVs (Brockmann et al., 2019). Overall, it is noteworthy that some synapses are prone to use more than one mode of priming.

Understanding how different synapses encode the incoming AP pattern into release requires studying the unique transduction apparatus expressed in individual synapses. We must dissect not only the individual role of molecules in the synapse but also how these molecules work together. For instance, our experiment examining the Munc13-1 concentration dependency of priming extends the previous studies (Deng et al., 2011) by showing that RIM boosts the Munc13-1 priming function by approximately fourfold. Therefore, we require more sophisticated models than single protein, loss-of-function experiments. In this study, we aimed to investigate how synapses respond to the relative changes in expression of AZ components by modifying the protein expression levels and studying the isoform-specific functions. We provide the first step in characterizing the role of RIM and Munc13 in small synapses to facilitate the understanding of complex molecular functions at the AZ.
