# Supplementary material for: Disentangling the Roles of RIM and Munc13 in Synaptic Vesicle Localization and Neurotransmission
Source: J Neurosci. 2020 Dec 2;40(49):9372–85. doi: 10.1523/JNEUROSCI.1922-20.2020 (PMC7724145; doi:10.1523/JNEUROSCI.1922-20.2020)
Supplement: Figure 1-1 — Values and statistics corresponding to Figure 1. Download Figure 1-1, DOCX file. [file ns-JN-RM-1922-20-s01.docx]

| Figure 1 | RIM^flox^ + ∆Cre | RIM^flox^ + Cre | *p*-value | Munc13-1 WT | Munc13-1 KO | *p*-value |
| --- | --- | --- | --- | --- | --- | --- |
| n/N | n= 153/3 | n = 161/3 |  | n = 148/3 | n = 173/3 |  |
| PSD length (nm) | 281.8 ± 12.8 | 286.7 ± 8.95 | 0.0939 | 312.3 ± 10.72 | 333.4 ±12.03 | 0.3821 |
| Docked SV | 1.10 ± 0.09 | 0.73 ± 0.071 | 0.0019 | 1.57 ± 0.10 | 0.56 ± 0.07 | <0.0001 |
| Proximal SV | 1.80 ± 0.12 | 1.28 ± 0.10 | 0.0004 | 2.03 ± 0.12 | 2.09 ± 0.13 | 0.8534 |
| Distal SV | 8.96 ± 0.35 | 8.60 ± 0.3 | 0.6746 | 9.61 ± 0.34 | 10.23 ± 0.38 | 0.6771 |
| n = number of synapses; N= number of cultures, Values indicate mean ± SEM, Unpaired t test (Mann-Whitney test) | | | | | | |

Figure 1-2. Values and statistics corresponding to Figure 1
