# Supplementary material for: Disentangling the Roles of RIM and Munc13 in Synaptic Vesicle Localization and Neurotransmission
Source: J Neurosci. 2020 Dec 2;40(49):9372–85. doi: 10.1523/JNEUROSCI.1922-20.2020 (PMC7724145; doi:10.1523/JNEUROSCI.1922-20.2020)
Supplement: Figure 2-1 — Values and statistics corresponding to Figure 2. Download Figure 2-1, DOCX file. [file ns-JN-RM-1922-20-s03.docx]

| Figure 2A, B | ∆Cre + Scr. | ∆Cre + M13-1 KD | Cre + Scr. | Cre + M13-1 KD | Test statistics |
| --- | --- | --- | --- | --- | --- |
| n/N | 3/3 | 3/3 | 3/3 | 3/3 |  |
| Norm. Munc13-1/Tubulin expression | 1 | 0.02 ± 0.009 | 0.35 ± 0.08 | 0.01 ± 0.002 | F (3, 8) = 133.8, *p* < 0.0001 |
| Norm. RIM/Tubulin expression | 1 | 0.70 ± 0.06 | 0.14 ± 0.09 | 0.11 ± 0.06 | F (3, 8) = 48.07, *p* < 0.0001 |
| n = number of repeats; N= number of cultures, Values indicate mean ± SEM, test: One way ANOVA | | | | | |
|  |  |  |  |  |  |
| Figure 2C-H | ∆Cre + Scr. | ∆Cre + M13-1 KD | Cre + Scr. | Cre + M13-1 KD | Test statistics |
| n/N | 157/3 | 148/3 | 168/3 | 167/3 |  |
| Docked SVs | 2.13 ± 0.11 | 1.42 ± 0.09 | 1.61 ± 0.1 | 0.95 ± 0.07 | H = 64.74, *p* < 0.0001 |
| Proximal SVs | 2.53 ± 0.13 | 2.95 ± 0.18 | 1.95 ± 0.13 | 1.98 ± 0.14 | H = 30.05, *p* < 0.0001 |
| Distal SVs | 12.69 ± 0.38 | 12.92 ± 0.45 | 12.45 ± 0.38 | 12.8 ± 0.36 | H = 1.928, *p* = 0.5874 |
| n = number of synapses; N= number of cultures, Values indicate mean ± SEM, H test: Kruskal-Wallis test | | | | | |

Figure 2-2. Values and statistics corresponding to Figure 2.
