# Supplementary material for: Disentangling the Roles of RIM and Munc13 in Synaptic Vesicle Localization and Neurotransmission
Source: J Neurosci. 2020 Dec 2;40(49):9372–85. doi: 10.1523/JNEUROSCI.1922-20.2020 (PMC7724145; doi:10.1523/JNEUROSCI.1922-20.2020)
Supplement: Figure 3-1 — Absolute values and statistics corresponding to Figure 3. Download Figure 3-1, DOCX file. [file ns-JN-RM-1922-20-s05.docx]

| Figure 3 | RIM^flox^ + ∆Cre | RIM^flox^ + Cre | *p*-value | M13-1 WT | M13-1 KO | *p*-value |
| --- | --- | --- | --- | --- | --- | --- |
| n/N | 41/5 | 51/5 |  | 27/3 | 35/3 |  |
| RRP (nC) | 0.7 ± 0.1 | 0.08 ± 0.01 | *p* < 0.0001 | 0.69 ± 0.09 | 0.04 ± 0.006 | *p* < 0.0001 |
| n/N | 59/5 | 56/5 |  | 32/3 | 46/3 |  |
| EPSC amplitude (nA) | 6.3 ± 0.72 | 0.18 ± 0.05 | *p* < 0.0001 | 7.1 ± 0.89 | 0.19 ± 0.03 | *p* < 0.0001 |
| n/N | 48/5 | 49/5 |  | 31/3 | 42/3 |  |
| mEPSC frequency (Hz) | 6.67 ± 0.72 | 1.48 ± 0.33 | *p* < 0.0001 | 8.62 ± 0.92 | 0.72 ± 0.23 | *p* < 0.0001 |
| n/N | 41/5 | 50/5 |  | 27/3 | 35/3 |  |
| Pvr (%) | 6.4 ± 0.56 | 1.11 ± 0.3 | *p* < 0.0001 | 8.08 ± 0.74 | 3.50 ± 0.63 | *p* < 0.0001 |
| n/N | 57/5 | 54/5 |  | 32/3 | 43/3 |  |
| PPR | 1.13 ± 0.05 | 1.63 ± 0.11 | *p* < 0.0001 | 1.13 ± 0.06 | 1.34 ± 0.06 | *p* = 0.01 |
| n = number of cells; N= number of cultures, Values indicate mean ± SEM, Unpaired t test (Mann-Whitney test) | | | | | | |

Figure 3-1. Absolute values and statistics corresponding to Figure 3
