# Supplementary material for: Disentangling the Roles of RIM and Munc13 in Synaptic Vesicle Localization and Neurotransmission
Source: J Neurosci. 2020 Dec 2;40(49):9372–85. doi: 10.1523/JNEUROSCI.1922-20.2020 (PMC7724145; doi:10.1523/JNEUROSCI.1922-20.2020)
Supplement: Figure 4-1 — Values and statistics corresponding to Figure 4. Download Figure 4-1, DOCX file. [file ns-JN-RM-1922-20-s06.docx]

Figure 4-1. Values and statistics corresponding to Figure 4.

| Figure 4 A-B | ∆Cre + Scr. | ∆Cre + 2×10^5^ IU | ∆Cre + 5×10^5^ IU | ∆Cre + 10×10^5^  IU | ∆Cre + 20×10^5^ IU | ∆Cre + 40×10^5^ IU | Cre + Scr. | test statistics |
| --- | --- | --- | --- | --- | --- | --- | --- | --- |
| n/N | 3/3 | 3/3 | 3/3 | 3/3 | 3/3 | 3/3 | 3/3 |  |
| Norm. Munc13-1/Tubulin expression | 1 | 0.35 ± 0.09 | 0.25 ± 0.04 | 0.1 ± 0.04 | 0.06 ± 0.02 | 0.05 ± 0.02 | 0.30 ± 0.06 | F (6, 14) = 46.79,  *p* < 0.0001 |
| n = number of repeats; N= number of cultures, Values indicate mean ± SEM, test: One way ANOVA | | | | | | | | |
|  |  |  |  |  |  |  |  |  |
| Figure 4 C-D | ∆Cre + Scr. | ∆Cre + 2×10^5^ IU | ∆Cre + 5×10^5^ IU | ∆Cre + 10×10^5^  IU | ∆Cre + 20×10^5^ IU | ∆Cre + 40×10^5^ IU | Cre + Scr. | test statistics |
| n/N | 30/3 | 30/3 | 31/3 | 31/3 | 32/3 | 34/3 | 30/3 |  |
| Norm. Munc13-1/VGLUT1 expression | 1 ± 0.06 | 0.33 ± 0.05 | 0.22 ± 0.02 | 0.12 ± 0.01 | 0.1 ± 0.01 | 0.11 ± 0.01 | 0.33 ± 0.03 | H = 128.2, *p* < 0.0001 |
| n = number of cells; N= number of cultures, Values indicate mean ± SEM, H test: Kruskal-Wallis test | | | | | | | | |
