# Supplementary material for: Disentangling the Roles of RIM and Munc13 in Synaptic Vesicle Localization and Neurotransmission
Source: J Neurosci. 2020 Dec 2;40(49):9372–85. doi: 10.1523/JNEUROSCI.1922-20.2020 (PMC7724145; doi:10.1523/JNEUROSCI.1922-20.2020)
Supplement: Figure 5-1 — Absolute values and statistics corresponding to Figure 5. Download Figure 5-1, DOCX file. [file ns-JN-RM-1922-20-s07.docx]

| Figure 5 | ∆Cre + Scr. | ∆Cre + 2×10^5^ IU | ∆Cre + 5×10^5^ IU | ∆Cre + 10×10^5^  IU | ∆Cre + 20×10^5^ IU | ∆Cre + 40×10^5^ IU | Cre + Scr. | test statistics |
| --- | --- | --- | --- | --- | --- | --- | --- | --- |
| n/N | 60/6 | 44/4 | 44/4 | 46/5 | 29/3 | 22/3 | 22/2 |  |
| RRP (nC) | 0.42 ± 0.05 | 0.40 ± 0.06 | 0.16 ± 0.02 | 1.15 ± 0.03 | 0.12 ± 0.02 | 0.09 ± 0.02 | 0.1 ± 0.02 | H = 93.2, *p* < 0.0001 |
| n/N | 60/6 | 44/4 | 44/4 | 46/5 | 29/3 | 22/3 | 22/2 |  |
| EPSC amplitude (nA) | 4.17 ± 0.44 | 4.04 ± 0.65 | 1.81 ± 0.37 | 1.40 ± 0.30 | 1.04 ± 0.19 | 0.82 ± 0.21 | 0.26 ± 0.09 | H = 89.7, *p* < 0.0001 |
| n/N | 51/6 | 42/4 | 39/4 | 40/5 | 29/3 | 20/3 | 17/2 |  |
| mEPSC frequency (Hz) | 4.95 ± 0.65 | 2.50 ± 0.43 | 2.65 ± 0.71 | 1.21 ± 0.34 | 1.42 ± 0.27 | 1.0 ± 0.26 | 0.15 ± 0.11 | H = 55.46, *p* < 0.0001 |
| n/N | 60/6 | 44/4 | 44/4 | 46/5 | 27/3 | 22/3 | 21/2 |  |
| Pvr (%) | 6.74 ± 0.66 | 5.58 ± 0.63 | 5.55 ± 0.65 | 5.4 ± 0.70 | 6.40 ± 1.06 | 4.39 ± 0.80 | 0.75 ± 0.15 | H = 51.42, *p* < 0.0001 |
| n = number of cells; N= number of cultures, Values indicate mean ± SEM, H test: Kruskal-Wallis test | | | | | | | | |

Figure 5-1. Absolute values and statistics corresponding to Figure 5.
