# Supplementary material for: Disentangling the Roles of RIM and Munc13 in Synaptic Vesicle Localization and Neurotransmission
Source: J Neurosci. 2020 Dec 2;40(49):9372–85. doi: 10.1523/JNEUROSCI.1922-20.2020 (PMC7724145; doi:10.1523/JNEUROSCI.1922-20.2020)
Supplement: Figure 6-1 — Absolute values and statistics corresponding to Figure 6. Download Figure 6-1, DOCX file. [file ns-JN-RM-1922-20-s08.docx]

| Figure 6A-G | ∆Cre + Scr. | ∆Cre + M13-1 KD | Cre + Scr. | Cre + M13-1 KD | Test statistics |
| --- | --- | --- | --- | --- | --- |
| n/N | 60/5 | 50/5 | 61/5 | 54/5 |  |
| RRP (nC) | 0.42 ± 0.04 | 0.08 ± 0.009 | 0.12 ± 0.03 | 0.05 ± 0.008 | H = 110.3, *p* < 0.0001 |
| n/N | 72/5 | 60/5 | 72/5 | 63/5 |  |
| EPSC amplitude (nA) | 4.06 ± 0.48 | 0.75 ± 0.12 | 0.23 ± 0.06 | 0.13 ± 0.04 | H = 159.1, *p* < 0.0001 |
| n/N | 62/5 | 55/5 | 64/5 | 56/5 |  |
| mEPSC frequency (Hz) | 5.34 ± 0.60 | 1.07 ± 0.22 | 0.50 ± 0.12 | 0.50 ± 0.34 | H = 97.37, *p* < 0.0001 |
| n/N | 60/5 | 50/5 | 58/5 | 47/5 |  |
| Pvr (%) | 6.19 ± 0.65 | 4.16 ± 0.44 | 0.8 ± 0.11 | 0.95 ± 0.29 | H = 112.6, *p* < 0.0001 |
| n = number of cells; N= number of cultures, Values indicate mean ± SEM, H test: Kruskal-Wallis test | | | | | |

Figure 6-1. Absolute values and statistics corresponding to Figure 6.
