# Supplementary material for: Disentangling the Roles of RIM and Munc13 in Synaptic Vesicle Localization and Neurotransmission
Source: J Neurosci. 2020 Dec 2;40(49):9372–85. doi: 10.1523/JNEUROSCI.1922-20.2020 (PMC7724145; doi:10.1523/JNEUROSCI.1922-20.2020)
Supplement: Figure 7-1 — Values and statistics corresponding to Figure 7. Download Figure 7-1, DOCX file. [file ns-JN-RM-1922-20-s09.docx]

| Figure 7A-B | ∆Cre + Scr. | ∆Cre + Munc13-1 KD | Cre + Scr. | Cre + Munc13-1 KD | Test statistics |
| --- | --- | --- | --- | --- | --- |
| n/N | 31/4 | 28/4 | 29/4 | 23/4 |  |
| RRP ratio (Suc2/Suc1) | 0.89 ± 0.03 | 1.33± 0.08 | 1.07 ± 0.08 | 1.45 ± 0.11 | H = 69.03, *p* < 0.0001 |
| n = number of cells; N= number of cultures, Values indicate mean ± SEM, H test: Kruskal-Wallis test | | | | | |
|  |  |  |  |  |  |
| Figure 7C-D | Scr. | Munc13-1 KD | *p*-value |  |  |
| n/N | 25/2 | 15/2 |  |  |  |
| RRP ratio (Suc2/Suc1) | 0.88 ± 0.06 | 0.99 ± 0.11 | 0.6991 |  |  |
| n = number of cells; N= number of cultures, Values indicate mean ± SEM, Unpaired t test (Mann-Whitney test) | | | |  |  |

Figure 7-1. Values and statistics corresponding to Figure 7
